# Generalizability of EEG-Based DEMENTIA Classifiers: A Multicenter study of Alzheimer’s, MCI, and FTD

**DOI:** 10.64898/2026.07.28.26359133

**Published:** 2026-07-29

**Authors:** Laouen Belloli, Nicolás Bruno, Hernan Hernandez, Jhosmary Cuadros, Damián Dellavale, Pavel Prado, Renato Anghinah, Bahar Güntekin, Lütfü Hanoğlu, Mario A Parra, Agustín Ibañez, Jacobo Sitt

**Affiliations:** 1 Institut du Cerveau, ICM, Inserm, CNRS, Sorbonne Université, Paris, France.; 2 ICC, CONICET & Universidad de Buenos Aires, Argentina.; 3 Instituto de Física Aplicada, CONICET & Universidad de Buenos Aires, Argentina.; 4 Latin American Brain Health Institute (BrainLat), Universidad Adolfo Ibáñez, Santiago, Chile.; 5 Global Brain Health institute, University of California San francisco, San francisco, USA.; 6 Global Brain Health Institute (GBHI), Trinity College Dublin (TCD), Dublin, Ireland.; 7 Cognitive Neuroscience Center (cnc), Universidad de San Andrés & CONICET, Buenos Aires, Argentina.; 8 Cognition Rehabilitation after TBI Center Universidade de Sao Paulo, São Paulo, Brazil.; 9 Department of Neurology, Faculdade de Medicina do ABC, Santo André, Brazil.; 10 Istanbul Medipol University, School of Medicine, Department of Biophysics, Türkiye.; 11 Brain and Cognition Research Center, BEYKOG, İstanbul Medipol University, Türkiye.; 12 Istanbul Medipol University, School of Medicine, Department of Neurology, Türkiye.; 13 Consejo Nacional de Investigaciones Científicas y Técnicas (CONICET), Centro Atómico Bariloche, San Carlos de Bariloche, Río Negro, Argentina.; 14 Escuela de Fonoaudiología, Facultad de Ciencias de la Rehabilitación y Calidad de Vida, Universidad San Sebastián, Santiago de Chile, Chile.; 15 Department of Psychological Sciences and Health, University of Strathclyde, Glasgow, UK.; 16 Advanced Center for Electrical and Electronic Engineering, Universidad Técnica Federico Santa María, Valparaíso 2390123, Chile.; 17 Grupo de Bioingeniería, Decanato de Investigación, Universidad Nacional Experimental del Táchira, San Cristóbal 5001, Venezuela.

**Keywords:** Alzheimer’s disease, mild cognitive impairment, frontotemporal dementia, EEG, machine learning, multi-center designs

## Abstract

EEG-based machine learning shows promise for neurodegenerative disease classification, but robustness to sample imbalance, center heterogeneity, and validation leakage remains a key concern for clinical translation. We developed a new framework to assess diagnostic performance, calibration, and cross-center generalizability of EEG multifeatured classifiers across CN (cognitively normal), MCI (mild cognitive impairment), AD (Alzheimer’s disease), and FTD (frontotemporal dementia), while addressing imbalance, statistical uncertainty, and validation rigor across six centers. Supervised classifiers were evaluated at aggregated- and subject-level repeated cross-validation and leave-one-center-out (LOCO) schemes, and calibration was implemented via Platt scaling within strictly nested folds. CN vs AD classification showed robust performance and cross-center generalizability, with consistent AUC and calibration across cross-validation and leave-one-center-out analyses. In contrast, CN versus MCI showed moderate, heterogeneous performance and limited cross-center generalizability, with chance-level results in some cohorts, while MCI versus AD showed moderate discrimination in a single available center. FTD contrasts showed modest or limited performance due to sparse samples. Predicted probabilities were stable across validation regimes for AD, but less consistent for MCI and FTD, and correlated robustly with cognitive impairment severity only for AD. Feature importance analyses identified disease-specific signatures, including alpha-band degradation and slow-wave increases in AD, with weaker and more heterogeneous patterns in prodromal and differential dementia contrasts (FTD vs AD). EEG classifiers provided robust discrimination for CN vs AD but showed limited and heterogeneous performance for MCI and FTD across centers. These results emphasize the need for balanced sampling, strict validation of clinical and EEG protocols, and uncertainty quantification to support reliable clinical deployment.

## 1 Introduction

Neurodegenerative dementias represent one of the most pressing healthcare challenges of the 21st century, with a substantial increase in projected global prevalence (GBD 2019 Dementia Forecasting Collaborators, 2022; Custodio et al., 2017; Livingston et al., 2020). This demographic transition disproportionately affects low- and middle-income countries, where exposure to risk factors including socioeconomic disparities, environmental pollutants, and infectious diseases accelerates brain aging (Legaz et al., 2025; Hernandez et al., 2025; Prado, Medel, et al., 2023). The implementation of early and affordable diagnostic strategies is vital in these settings, as timely detection provides significant health-economic benefits and improves clinical outcomes (Kwon et al., 2025). Mainstream diagnostic frameworks have traditionally relied on pathological biomarkers such as amyloid beta and tau proteins, quantified through positron emission tomography (PET) or cerebrospinal fluid (CSF) analysis (Jack et al., 2018; McKhann et al., 2011; Dubois et al., 2014). While these methods offer high sensitivity, their clinical utility in global settings is hindered by prohibitive costs and extremely limited access, particularly in developing economies (Parra et al., 2021; Parra et al., 2023; Custodio et al., 2024; Duran-Aniotz et al., 2021). Although blood-based (plasma) biomarkers have emerged as a promising alternative (Ntymenou et al., 2021), they are not yet widely accessible and lack systematic validation in populations (Parra et al., 2018; Coronel-Oliveros et al., 2024). Moreover, the accuracy of these markers in diverse populations can be influenced by whole-body health (Ibáñez et al., 2025), and usually requires the combination of neuroimaging and cognitive assessments (Caviedes et al., 2026).

Electroencephalography (EEG) emerges as a cost-effective complement, also providing a rich characterization of temporal brain dynamics (Rossini et al., 2020). EEG is uniquely scalable for global initiatives, being non-invasive, portable, low-cost, and widely available across clinical settings (Whelan et al., 2022). This technique has identified multiple neurophysiological signatures of neurodegeneration, such as spectral slowing, reduced signal complexity, and network disconnection observed in Alzheimer’s disease (Moguilner et al., 2024; Prado, Mejía, et al., 2023; Babiloni et al., 2021; Dauwels et al., 2011). Furthermore, distinct oscillatory, topographic and mechanistic patterns differentiate dementia subtypes, such as Alzheimer’s, frontotemporal dementia and mild cognitive impairment (Coronel-Oliveros et al., 2025; Jelic et al., 2000; Nishida et al., 2011). These markers can feed automated classification algorithms, demonstrating good diagnostic accuracy by exploiting multivariate patterns (Akbar et al., 2025; Yuan & Zhao, 2025; Moguilner et al., 2022). Thus, computational multifeature EEG architectures can help characterize dementia in global settings.

Despite this progress, several factors could hinder the clinical translation of EEG-based biomarkers. Prior studies are frequently constrained by methodological weaknesses, including small sample sizes, potential data leakage, and insufficient statistical validation, which may inflate performance estimates and undermine reproducibility. Moreover, technical, demographic, and site-specific heterogeneity (Prado et al., 2022) spanning EEG acquisition systems, preprocessing pipelines, and population diversity introduces variance that may obscure disease-specific signals and compromise robustness in diverse cohorts (Moguilner et al., 2024). Existing work usually lacks rigorous multi-center validation using leave-one-center-out or similarly stringent frameworks (Prado, Mejía, et al., 2023; Peh et al., 2021), leaving generalizability largely unproven. Beyond technical performance, most approaches remain poorly aligned with clinical decision-making, relying on binary classifications rather than calibrated probabilistic outputs anchored in neuropsychological standards. These approaches often lack interpretability regarding the neurophysiological features driving predictions, limiting clinical relevance and regulatory adoption (Malik et al., 2025). Finally, much of the literature focuses narrowly on distinguishing Alzheimer’s disease from cognitively normal controls, with insufficient attention to clinically essential challenges (Odusami et al., 2024) such as differential diagnosis necessary for meaningful clinical deployment (Rossini et al., 2020).

**Figure 1:**
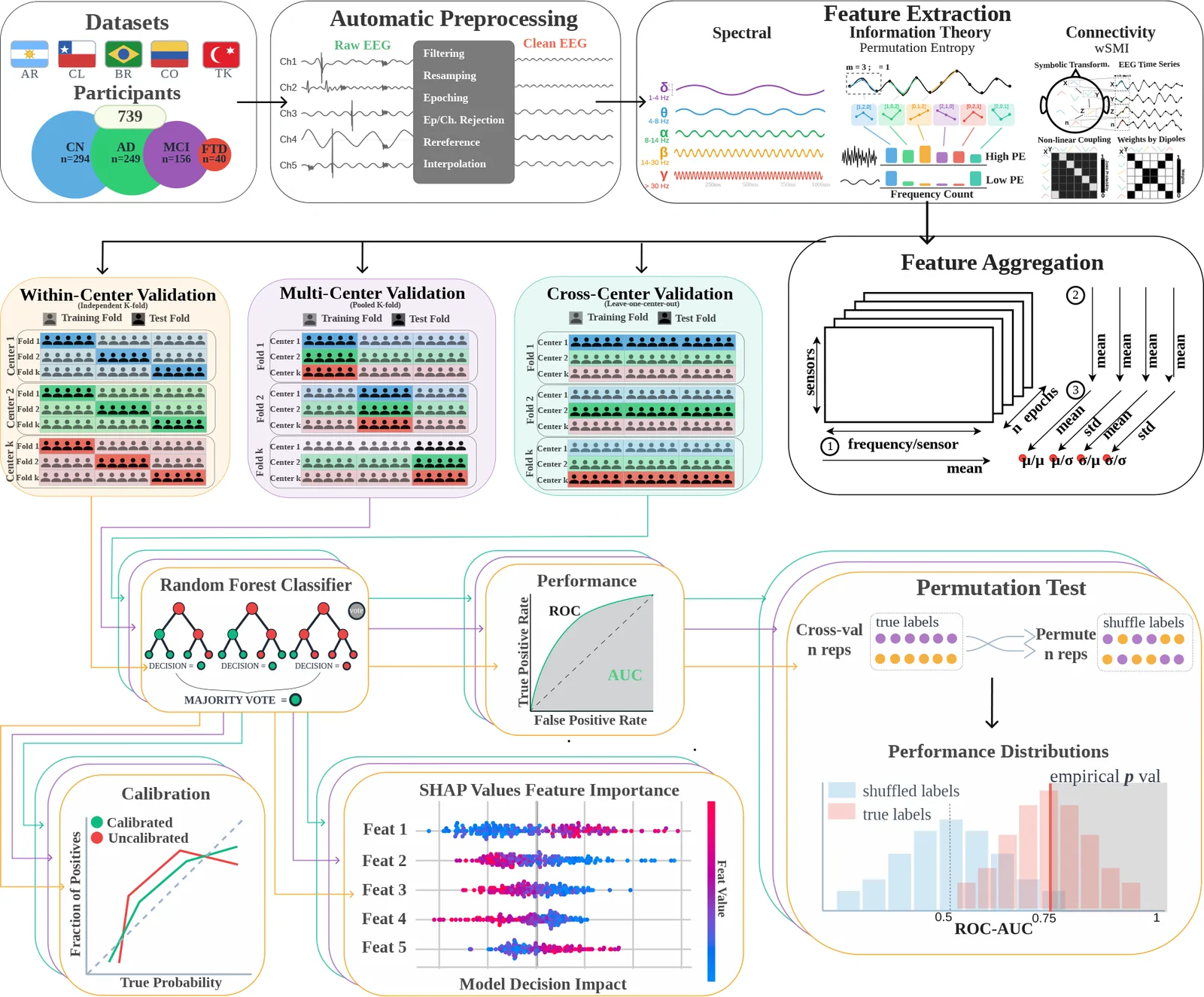
Comprehensive EEG-based machine learning pipeline for dementia classification across multiple clinical centers. The pipeline encompasses five major components: Datasets: Multi-center cohort including 739 participants across five sites (Argentina, Colombia, Brazil, Chile, Turkey) with four diagnostic groups: healthy controls (CN, n=294), Alzheimer’s disease (AD, n=249), mild cognitive impairment (MCI, n=156), and frontotemporal dementia (FTD, n=40). Automatic Preprocessing: Standardized EEG processing including filtering, downsampling, epoching, artifact rejection, re-referencing and interpolation using clean EEG protocols. Feature Extraction: Extraction of 92 neurophysiological features across three domains: spectral markers (frequency band powers), information theory measures (permutation entropy, Kolmogorov complexity), and connectivity indices (weighted symbolic mutual information) following the Sitt et al. framework. Feature Aggregation: Dual reduction approach computing spatial (mean and global field power across electrodes) and temporal (80% trimmed mean and standard deviation across epochs) statistics, yielding four feature subtypes (*μ/μ, σ/μ, μ/σ, σ/σ*) per biomarker. Machine Learning: Random Forest classification with and without probability calibration using cross-validation. Three-tier Validation: Single-center (within-site), multi-center (pooled), and cross-center (leave-one-site-out) validation strategies to assess model generalizability. Performance Evaluation: ROC-AUC analysis, reliability diagrams comparing calibrated vs. uncalibrated predictions, and statistical validation using permutation testing. Model Interpretability: SHAP values analysis for feature importance ranking and decision impact assessment. This comprehensive framework enables robust assessment of EEG biomarkers for dementia classification while addressing key translational challenges including cross-site generalizability and clinical interpretability.

We aimed to bridge these gaps through a systematic multi-center design spanning five independent cohorts across diverse cultural and geographical settings. We implemented a “plug-and-play” automated framework that prioritized automaticity to eliminate subjectivity and enhance generalizability across clinical settings. We included complementary domains (from spectral markers and signal complexity to inter-regional connectivity), to capture the multifaceted nature of neural dysfunction (Sitt et al., 2014; King et al., 2013). Although this framework had been previously applied in dementia research, those studies relied on reduced single-center designs with homogeneous cohorts (Gaubert et al., 2019, 2021), leaving its robustness across heterogeneous, multi-site settings untested. This framework has also been employed successfully in multicentric disorders of consciousness research (Sitt et al., 2014; Engemann et al., 2018; Pérez et al., 2024; Manasova et al., 2024) and sleep research (Strauss et al., 2022; Türker et al., 2023). A key advantage of this framework is its potential to maintain stable predictive accuracy even in low-density channel configurations, which supported its feasibility for resource-limited or purely clinical contexts. To rigorously evaluate model generalization, we implemented a three-tier validation strategy: single-center repeated stratified k-fold cross-validation, pooled multi-center stratified k-fold cross-validation, and leave-one-center-out cross-validation as the gold standard for assessing cross-site robustness. Furthermore, our clinical evaluation extended beyond AD versus CN comparisons to include CN versus MCI for early detection, MCI versus AD for disease stage, and FTD versus AD for differential diagnosis. Finally, we incorporated calibrated probability estimation to generate scores that could be directly interpreted in clinical decision-making (Niculescu-Mizil & Caruana, 2005), alongside SHAP-based feature importance to identify center-independent EEG biomarkers with the highest relevance (Figure 1). We hypothesized that multifeature EEG classifiers would demonstrate significant cross-center generalizability despite heterogeneous acquisition conditions, with performance decreasing as validation stringency increases. A graded discriminative pattern should present highest accuracy for CN vs AD, intermediate performance for MCI-related contrasts, and lower accuracy for differential diagnosis between dementia syndromes due to greater neurophysiological overlap. EEG-derived probabilistic predictions would show clinically meaningful associations with cognitive impairment and rely on physiologically interpretable biomarkers consistent with known neurodegenerative mechanisms.

## 2 Methods

### 2.1 Datasets

The current study utilized resting-state EEG data collected from five independent centers in Latin America and Europe, comprising a total of 739 participants (61% females): 294 cognitively normal (CN) individuals, 156 patients with mild cognitive impairment (MCI), 249 with Alzheimer’s disease (AD), and 40 with frontotemporal dementia (FTD), see Table 1 for the distribution between the different centers. All recordings corresponded to eyes-closed resting-state protocols. Despite differences in hardware and acquisition systems, all datasets were harmonized through a standardized preprocessing pipeline involving band-pass filtering, resampling, epoching, bad-channels and bad-epochs rejection, average reference, and bad-channel interpolation as detailed below.

Participants were recruited from centers in Argentina (*BRAINLAT*_*ar*_), Chile (*BRAINLAT*_*cl*1*/*2_), Turkey (Izmir University of Economics, *IUEMF*_*tr*_), Colombia (Universidad Surcolombiana, *USCO* – *UCC*_*co*_), and Brazil (Universidade de São Paulo, *USP*_*br*_). The distribution of participants across diagnostic categories and centers is presented in Table 1. Diagnostic classification followed international consensus criteria: MCI was defined according to the Petersen criteria with a Mini-Mental State Examination (MMSE) score ≥ 24; AD diagnosis fulfilled the NINCDS–ADRDA criteria for probable AD; and FTD diagnosis met the revised international consensus criteria for probable behavioral-variant FTD. All participants provided written informed consent in accordance with the Declaration of Helsinki, and all protocols were approved by the respective institutional ethics committees: INECO–San Martín de Tours (FWA00028264, Argentina); Hospital Clínico Universidad de Chile (FWA00029089, Chile); Universidad Adolfo Ibáñez (UAI) and Universidad de Santiago de Chile (USACH) for the *BRAINLAT*_*cl*2_ cohort; Universidade de São Paulo Hospital das Clínicas (FWA00001035, Brazil); Universidad de Antioquia (FWA00028864, Colombia); Hospital Universitario Hernando Moncaleano Perdomo (Bioethics and Research Committee No. 002–006, Colombia); and the Izmir University of Economics Ethics Committee (Decision 2018/05–09, Protocol 3815-GOA, Turkey).

EEG acquisition parameters for each site are summarized below:

- *BRAINLAT*_*ar*_: 128-channel Biosemi ActiveTwo AdBox Mk2 system, 2048 Hz sampling rate, Biosemi montage with linked mastoid reference.
- *BRAINLAT*_*cl*1_: 128-channel Biosemi ActiveTwo AdBox Mk2 system, 2048 Hz sampling rate, Biosemi montage with linked mastoid reference.
- *BRAINLAT*_*cl*2_: 128-channel Biosemi ActiveTwo system, 1024 Hz sampling rate, Biosemi montage with linked mastoid reference.
- *IUEMF*_*tr*_: 32-channel BrainAmp system, 500 Hz sampling rate, standard 10–20 montage.
- *USCO* – *UCC*_*co*_: 64-channel Biosemi ActiveTwo system, 1024 Hz sampling rate, Biosemi montage with linked mastoid reference.
- *USP*_*br*_: 21-channel EEGLAB system, 200 Hz sampling rate.

### 2.2 EEG preprocessing

To ensure cross-center comparability, all recordings were processed using a fully automated and harmonized pipeline implemented in MNE-Python (Gramfort et al., 2014) and the NICE framework (Sitt et al., 2014; Engemann et al., 2018). Non-EEG channels were removed, and signals were band-pass filtered (0.5–45 Hz) and resampled to 250 Hz to standardize the temporal resolution of frequency-dependent markers across acquisition systems. Continuous data were segmented into fixed-length epochs, followed by automated rejection of bad channels and epochs based on amplitude and variance criteria. An average reference was applied, and rejected channels were reconstructed through spherical spline interpolation to preserve montage geometry.

This standardized workflow was designed to minimize hardware- and montage-dependent variability while retaining neurophysiological information relevant for the 23 EEG markers. Data quality was comparable across sites, with mean bad channels ranging from 1.36–5.69% and bad epochs from 0.98–15.07%, and all recordings meeting predefined inclusion criteria. Signal quality was further verified using the Overall Data Quality (ODQ) index (Zhao et al., 2023), confirming no systematic differences in artifact contamination across diagnostic groups (Supplementary Methods 10.5.9). Full preprocessing specifications and quality-control criteria are provided in Supplementary Methods 10.5.

### 2.3 EEG feature extraction

Twenty-three EEG biomarkers were extracted following the framework of Sitt et al. (2014) and Engemann et al. (2018), which combines complementary descriptors of neural dynamics. Markers were organized into three families (Supplementary Table S2, Figure 1: Feature Extraction): spectral measures, indexing oscillatory slowing and power distribution; information-theoretic measures (permutation entropy and Kolmogorov complexity) capturing signal irregularity; and connectivity measures based on weighted symbolic mutual information (wSMI) to quantify nonlinear inter-regional coupling while reducing volume-conduction effects.

To obtain representations robust to montage and hardware differences, each marker was summarized along two dimensions. Across channels, we computed the mean activity and its spatial standard deviation, the latter reflecting global field power (i.e., how heterogeneous the marker is over the scalp). Across epochs, we used the 80% trimmed mean to capture typical activity and the standard deviation to quantify temporal variability. Combining these spatial and temporal summaries produced four intuitive descriptors: average activity, global field power, temporal variance, and joint spatiotemporal variability, yielding 92 feature subtypes in total.

Feature extraction was performed using identical parameters for all centers. Detailed computational definitions, frequency bands, and aggregation formulas are provided in Supplementary Methods 10.6.

### 2.4 Classification Procedure

The classification model was implemented as a scikit-learn pipeline combining a StandardScaler and a RandomForest-Classifier. While feature scaling is not required for tree-based models, its inclusion ensures consistency across potential model comparisons. The Random Forest classifier was selected due to its robustness in high-dimensional settings, as it performs implicit feature selection through random subspace sampling and reduces variance via bootstrap aggregation.

Importantly, no hyperparameter optimization was performed, and default model parameters were used throughout. This design choice was made to minimize model selection bias and reduce the risk of overfitting to specific cross-validation splits, particularly given the heterogeneity and limited sample sizes typical of multi-center clinical datasets. Rather than maximizing predictive performance, our goal was to assess the stability and generalizability of the extracted features under a minimally tuned and reproducible modeling framework.

Within this framework, three complementary validation levels were implemented to characterize performance under increasingly demanding generalization conditions (Figure 1 Within-, Multi- and Cross-Center Validation):

- **Within-center validation** assessed classification performance within each center independently to establish baseline discriminative capacity under controlled conditions. For each center containing multiple diagnostic categories, we applied Stratified by diagnostic category K-Fold cross-validation (k = 5 splits) to evaluate how well EEG markers distinguished diagnostic categories within homogeneous technical and demographic settings. This approach provided an estimate of classification performance by eliminating inter-site variability while preserving the fundamental challenge of distinguishing between diagnostic categories.
- **Multi-center validation** assessed model robustness across pooled heterogeneous datasets to evaluate performance under realistic data diversity. Data from multiple centers were combined and evaluated through Stratified by diagnostic category K-Fold cross-validation (k = 5 splits) at the patient level, allowing models to train and test on mixed populations while experiencing technical and demographic heterogeneity within each fold. This approach introduced variability from differences in EEG equipment, channel configurations, and participant demographics. The key distinction from single-center validation was that models experienced cross-site variability during training, enabling adaptation to heterogeneous conditions while maintaining exposure to all contributing centers during the training phase. Nevertheless, because the model was trained with data from the same centers on which it was evaluated, it was not blind to those technical and demographic conditions, allowing the model to learn the center’s effect. Although it learned to be robust across setups, it was not evaluated under complete generalization to unseen conditions. This validation could only be applied to diagnostic comparisons with a given contrast available from at least two different sites.
- **Cross-center generalization** evaluated model transferability to completely unseen acquisition conditions using Leave-One-Center-Out (LOCO) validation to assess external validity. In each fold, one center was entirely excluded from training and used exclusively for testing. This represented the most stringent level of generalization challenge, as models were required to transfer knowledge acquired from specific technical and demographic contexts to entirely different environments. The number of folds was constrained by the number of participating centers, with classification tasks limited to diagnostic pairs available across multiple sites. This validation strategy directly addressed the fundamental question of model generalizability across unseen heterogeneous multicentric conditions.

This hierarchical evaluation scheme allowed the study to acknowledge data-availability limitations while still providing a rigorous demonstration of generalizable performance where the evidence permitted, and a transparent delineation of scenarios in which results remained preliminary. Each validation strategy addressed progressively more stringent generalization requirements: single-center validation established fundamental biomarker discriminative capacity, multi-center validation assessed robustness to heterogeneity with adaptation opportunity, and cross-center validation evaluated true external validity transfer across independent datasets, constituting strong evidence that the 23-marker representation captures disease-related neurophysiology rather than center-specific artifacts.

### 2.5 Model evaluation, calibration, and clinical validity analyses

To assess performance stability and statistical relevance, we employed a repeated resampling and permutation framework. Specifically, 500 repetitions of stratified K-Fold-/LOCO- validation were used to estimate the distribution of performance metrics, including ROC-AUC, precision, sensitivity, and confusion matrix components (TN, FP, FN, TP), reported as *μ* ± *σ*. In parallel, 500 repetitions with permuted labels were used to generate null distributions of ROC-AUC under the hypothesis of no association between features and labels.

Effect size was quantified as the difference between the mean ROC-AUC obtained with true labels and the mean ROC-AUC obtained under permutation (*μ*(*AUC*_*real*_) – *μ*(*AUC*_*perm*_)). Statistical significance (*p*_*val*_) was estimated empirically as the proportion of permuted ROC-AUC values exceeding the mean ROC-AUC obtained with true labels, providing a non-parametric measure of how unlikely the observed performance is under the null distribution. Finally, FDR correction was applied over the the obtained *p*_*val*_ to get the final significance *p*_*val*_ for each validation strategy, classification contrast and center.

To evaluate the reliability of predicted probabilities, we compared uncalibrated outputs with probabilities calibrated using Platt scaling within a five-fold cross-validation framework. To obtain stable estimates of calibration performance, predicted probabilities from all 500 repetitions were pooled. This was necessary to ensure sufficient sample size for the estimation of calibration curves.

Calibration performance was quantified using mean squared error (MSE)-based metrics. Specifically, we computed *Cal*_*err*_ as the MSE between the uncalibrated predicted probabilities and the ideal perfectly calibrated probabilities. We further quantified the benefit of calibration as *Cal*_Δ_, defined as the reduction in MSE achieved by calibration, computed as the difference between the MSE of the uncalibrated and calibrated predictions.

To assess the stability of individual-level predictions across validation regimes, we compared model-predicted probabilities obtained under within-center, multi-center, and cross-center validation. For each classification contrast, the subject-level predicted probabilities corresponding to out-of-fold test samples were averaged across repeated cross-validation iterations. Then, Spearman rank correlations were computed between pairs of validation strategies at the subject level to quantify the consistency of subject ranking across training conditions. Correlations were evaluated separately for each contrast and, when applicable, within centers. Statistical significance was assessed using the *p*_*val*_ obtained from the Spearman rank statistic, and FDR correction using the Benjamini–Hochberg procedure was applied across all correlations to account for multiple comparisons.

To evaluate whether EEG-based predictions captured clinically meaningful variation beyond demographic effects, we assessed associations between model-predicted probabilities and neuropsychological measures after demographic adjustment. For each diagnostic contrast and validation strategy, subject-level predicted probabilities corresponding to out-of-fold test samples were averaged across cross-validation repetitions. Both predicted probabilities and cognitive scores were residualized with respect to age, sex, and years of education using linear regression. Spearman rank correlations were then computed between residualized predictions and residualized cognitive measures. Analyses were performed separately for each contrast and validation strategy, and statistical significance was assessed with FDR correction using the Benjamini–Hochberg procedure across validation strategies, cognitive measures, and contrasts.

Model interpretability was assessed using SHAP values computed in an out-of-fold manner alongside the cross-validation procedure. For each fold, models were trained on the training partition, and SHAP values were estimated on the corresponding held-out test partition using a permutation-based explainer. The background distribution was defined using the training data of each fold, ensuring that explanations were computed without information leakage.

This procedure yielded, for each validation strategy, diagnostic contrast, sample, and feature, a distribution of SHAP values across 500 repetitions. SHAP values were subsequently averaged across repetitions for each sample and classification contrast to obtain robust estimates of feature importances.

To visualize the effect of individual markers, we examined the ten features with the highest absolute mean SHAP values for each classification task, analyzing how their magnitude related to SHAP contribution—that is, whether higher or lower feature values increased the likelihood of predicting one class over another. Subsequently, the five most influential features per task were all pooled together in a radar plot to facilitate cross-task comparison.

In addition to the main analysis, we performed a sanity check procedure to rule out possible dataset and class size imbalance by downsampling all datasets and classes to the smaller available size (16) and confirmed that effects are mostly maintained (Full explanation in Supplementary Section 10.1).

For a detailed explanation regarding the model validation steps please refer to Supplementary Section 10.7.

## 3 Results

### 3.1 Robust cross, multi-center generalization

We first evaluated whether EEG-based markers support reliable diagnostic discrimination under increasingly stringent generalization conditions, spanning within-center, multi-center, and cross-center validation. *Within-center* validation assesses discrimination when training and testing are performed within the same clinical site, reflecting performance under homogeneous recording and population conditions. *Multi-center* validation evaluates robustness when models are trained and tested on pooled data from multiple centers, exposing them to heterogeneous acquisition setups and demographics during training. Finally, *cross-center* validation tests true external generalization by evaluating models on an entirely unseen center, requiring transfer to new technical and population contexts. Together, these complementary strategies allow discrimination performance, robustness to heterogeneity, and cross-site transferability to be examined separately (Section 2).

**Figure 2:**
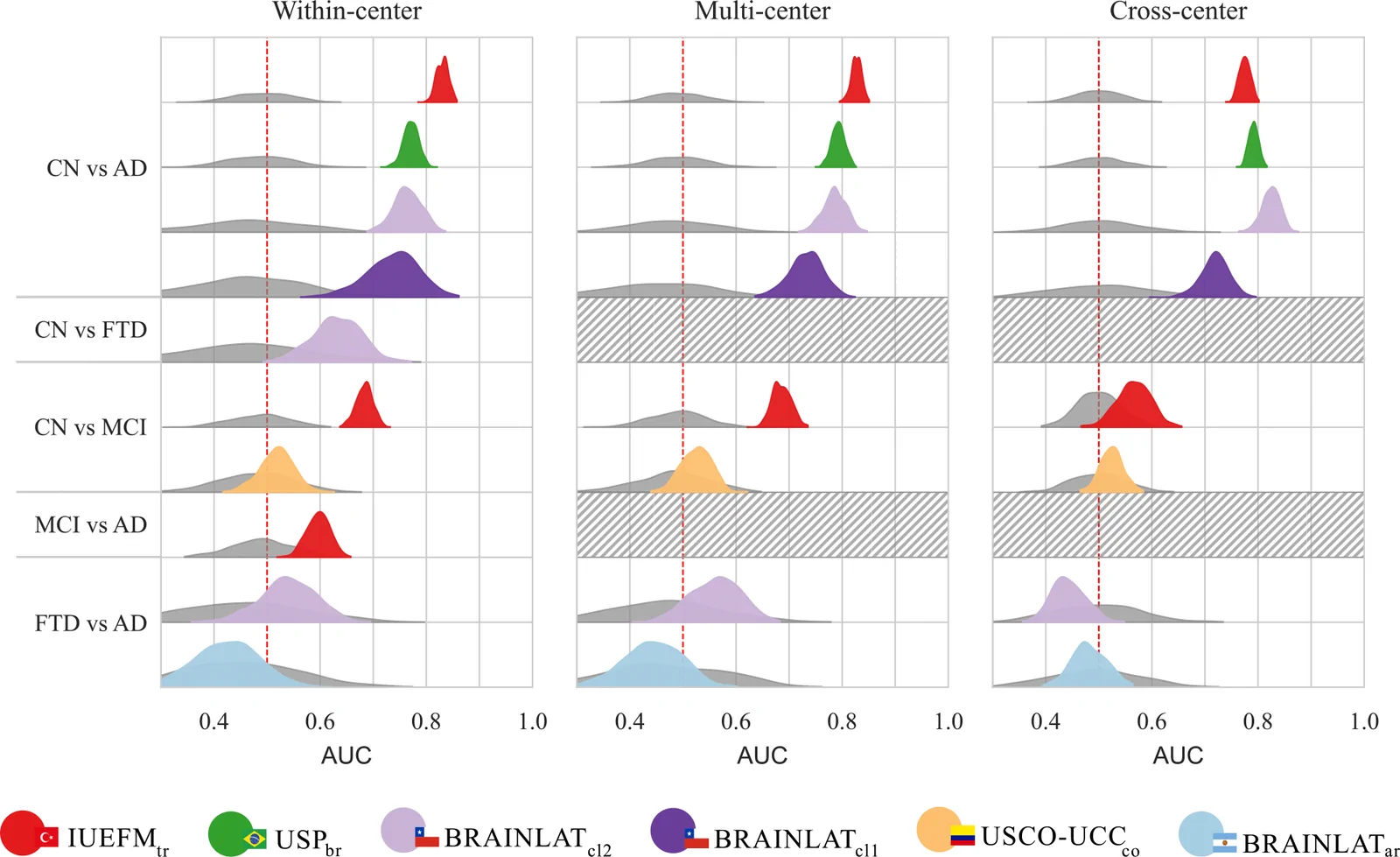
Classification performance across three validation strategies and five diagnostic comparisons. Each panel represents a different validation approach: *Within-center* (left) shows performance using Stratified by diagnostic category K-Fold cross-validation within individual centers; *Multi-center* (middle) displays results from pooled data across multiple sites using Stratified K-Fold cross-validation; *Cross-center* (right) illustrates generalization performance using Leave-One-Center-Out cross-validation, where each center was iteratively held out as an independent test set. Plots show the distribution of AUC scores across 500 repeated cross-validation iterations for each of five binary classification tasks (y-axis): CN vs AD, CN vs FTD, CN vs MCI, MCI vs AD, and FTD vs AD. Colors correspond to different acquisition centers/datasets: *BRAINLAT*_*ar*_ (light blue), *BRAINLAT*_*cl*1_ (dark purple), *BRAINLAT*_*cl*2_ (purple), *IUEFM*_*tr*_ (red), *USCO* – *UCC*_*co*_ (orange), *USP*_*br*_ (green). Hatched areas indicate diagnostic comparisons not available at particular centers due to absence of required diagnostic categories. The red dashed line at AUC = 0.5 marks chance-level performance.

The discrimination between CN individuals and AD patients revealed robust EEG signatures across all validation strategies (Figure 2, Table 2). Performance within individual centers demonstrated strong classification across all sites (AUC 0.74–0.83). These within-center results indicated that EEG markers captured clinically relevant differences within homogeneous recording environments. Exposure to heterogeneous data during training through multi-center validation showed maintained performance across sites. This pattern suggested that diverse recording conditions during training strengthened model robustness. The most stringent cross-center validation confirmed cross-site transferability, with performance remaining largely stable across centers. Performance showed a non-significant increase between multi- and cross-center validations for *BRAINLAT*_*cl*2_, the smallest dataset, and a non-significant decrease for *IUEFM*_*tr*_, the largest dataset, suggesting that minor performance variations were primarily driven by differences in dataset size rather than limited model generalizability. Permutation testing confirmed that classification performance was significantly above chance at every center, indicating that predictions were not driven by site-specific artifacts. Probability estimates were also well calibrated across centers and validation strategies.

The detection of MCI proved markedly more challenging than established dementia. Within-center analyzes showed only moderate discrimination, with substantial heterogeneity across sites, indicating that prodromal neural signatures were less consistent than those observed for CN vs AD (Figure 2, Table 2). Multi-center validation maintained the same discrimination performance, further highlighting population-specific variability in early disease expression, but with a marker signature consistent across sites. The most stringent cross-center validation further highlighted the limited generalizability of MCI detection, with performance remaining at chance level for *USCO* – *UCC*_*co*_ and reaching only modest discrimination for *IUEFM*_*tr*_. Importantly, *USCO* – *UCC*_*co*_ already exhibited near-chance performance in the within-center setting (*AUC* = 0.52), whereas training on *USCO* – *UCC*_*co*_ and testing on *IUEFM*_*tr*_ resulted in improved discrimination (*AUC* = 0.57). This finding indicates that informative patterns were present in the *USCO* – *UCC*_*co*_ data, but that group separation was less pronounced within this cohort, consistent with greater heterogeneity or less distinct clinical phenotypes. Although models often achieved high sensitivity, this occurred alongside modest overall discrimination, indicating limited underlying separability rather than robust classification. Calibration quality also varied considerably between centers. Together, these findings indicate that EEG markers capture weaker and more heterogeneous neurophysiological alterations at the MCI stage.

Distinguishing between MCI and AD disease was evaluated only at *IUEFM*_*tr*_ as we had no MCI vs AD data from the remaining centers. The within-center validation approach yielded moderate discrimination with AUC of 0.60 but statistically robust, reflecting the more challenging differentiation between advanced prodromal states and early dementia compared to CN vs AD (Figure 2, Table 2). Calibration residual error was 0.05 with improvement delta of 0.006, indicating that predicted probabilities required modest adjustment.

Distinguishing CN individuals from FTD patients was evaluated only at *BRAINLAT*_*cl*2_ as we had no CN vs FTD data from the remaining centers. Analysis within this single center achieved moderate performance with AUC of 0.63 compared with CN vs AD but statistically robust above chance level (Figure 2, Table 2). Calibration residual error was elevated at 0.02 with no significant calibration improvement, suggesting moderate deviation from optimal probability alignment. The absence of this comparison at other centers precluded multi-center and cross-center validation.

Differentiation between FTD and AD showed limited discriminative performance for *BRAINLAT*_*cl*2_ in both within- and multi-center validation strategies, while no discriminative capacity was observed for *BRAINLAT*_*ar*_ (Figure 2, Table 2).

To further assess whether the observed effects could be influenced by imbalances in sample size across diagnostic categories and centers, we conducted an additional analysis using a repeated stratified downsampling procedure (see Supplementary Material, Section 10.1). This approach enforces identical sample sizes across all diagnostic-by-center combinations prior to model training and evaluation. The results of this analysis were consistent with the main findings (Supplementary Figure S1), indicating that the performance patterns reported above are not driven by class or center imbalance.

### 3.2 Stability of individual-level predictions across validation regimes

**Figure 3:**
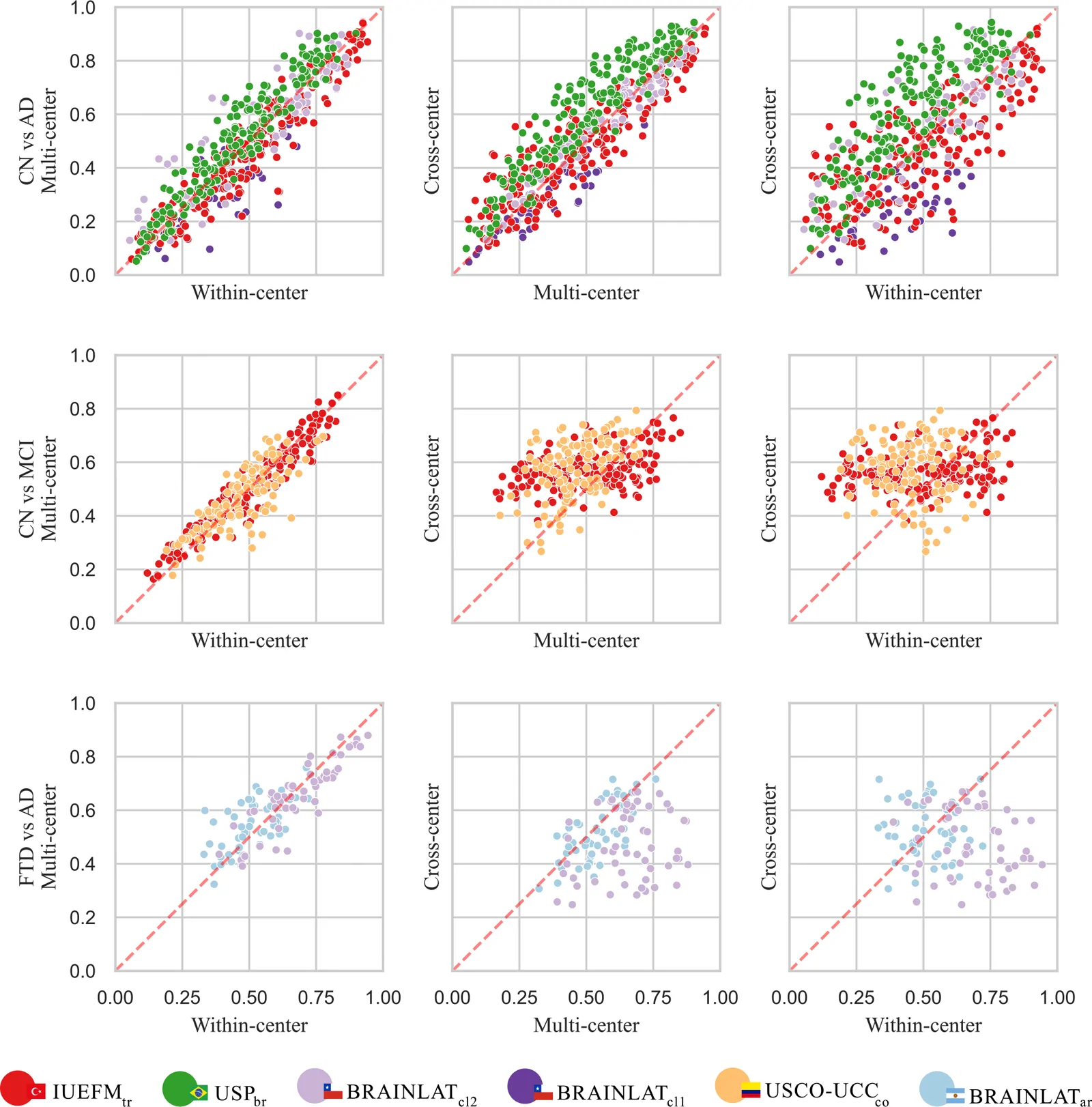
Spearman correlation matrices between paired validation strategies. Each scatter plot illustrates the relationship between predicted probabilities derived from two distinct validation strategies (columns), evaluated across different diagnostic contrasts (rows). Each point represents the mean predicted probability across repetitions for an individual participant, colored by acquisition center to visualize site-specific agreement. Spearman correlation coefficients (*ρ*) were computed for every pairwise comparison, both overall and stratified by center, to quantify the stability of decision boundaries under varying training regimes. High correlations indicate that the model maintains consistent ranking of participants regardless of the validation strategy, while low correlations suggest sensitivity to the training configuration. Complete center-specific correlation values are reported in Supplemental Table S3.

Beyond aggregate performance metrics, we examined whether individual-level predicted probabilities remained stable across validation regimes, thereby assessing the consistency of subject ranking under changing training conditions. For CN vs AD, predicted probabilities were highly concordant between within-, multi-, and cross-center validation strategies (Figure 3, Supplemental Table S3), indicating that participants were ranked similarly even when the model was exposed to unseen sites. This suggests that decision boundaries for established dementia relied on robust, center-independent EEG patterns. In contrast, CN vs MCI and FTD vs AD comparisons showed markedly reduced agreement when cross-center generalization was required. While within- and multi-center models produced consistent rankings, correlations involving the cross-center strategy dropped substantially, revealing more sensitivity to domain shift. These findings parallel the limited cross-center performance for these contrasts.

### 3.3 EEG-derived predictions align with cognitive impairment clinical tests beyond demographic effects

We first examined associations between predicted probabilities and a broad set of neuropsychological tests to characterize their overall relationship with cognitive impairment. A comprehensive analysis of these unadjusted associations, which exhibit stronger effect sizes, is provided in Supplementary Material 10.2.

**Figure 4:**
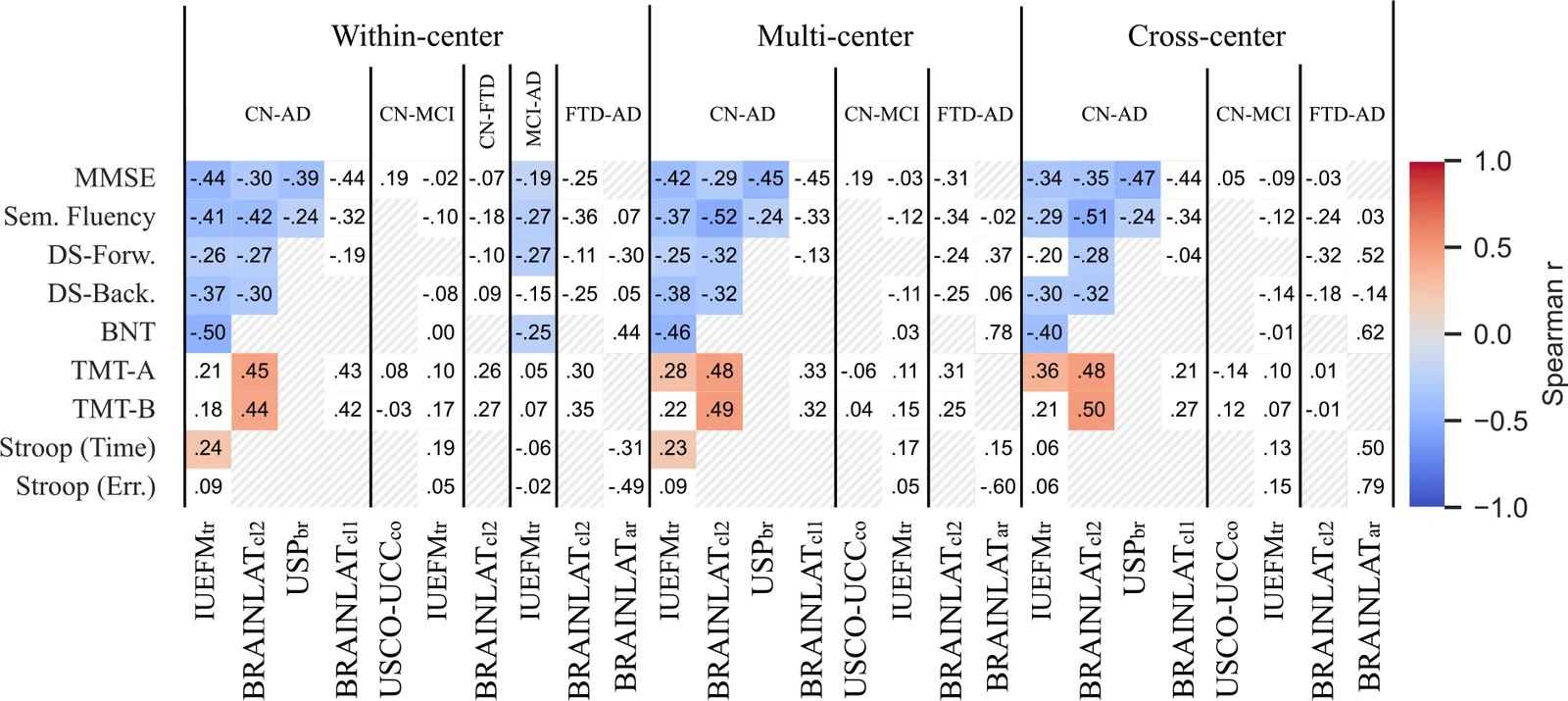
Partial Spearman correlations between model-predicted probabilities and cognitive test performance after controlling for demographic variables (age, sex, education). Each column represents a specific classification task (CN-AD, CN-MCI, CN-FTD, MCI-FTD, FTD-AD) within three validation approaches: within-center (left panel), multi-center (middle panel), and cross-center (right panel). Model predictions ranged from 0 (first class) to 1 (second class) in each binary comparison, with lower performance indicating greater impairment. Correlation coefficients are displayed within cells, with color intensity representing magnitude and direction (blue: negative, red: positive). Non colored cells indicate non significant p-values after FDR correction, while hatched cells indicate non available data. After accounting for demographic influences, negative correlations between predicted probabilities and cognitive performance (MMSE, Semantic Fluency, Digit Span, Boston Naming Test) persisted across validation schemes, demonstrating that model predictions captured disease-related cognitive decline independent of demographic factors. Positive associations with Trail Making Test remained evident, reflecting executive function deficits associated with neurodegenerative progression. The persistence of these associations after demographic adjustment suggests that EEG-derived predictions reflected genuine pathological processes rather than confounding demographic influences.

Here, we focus on a more stringent analysis in which associations between predicted probabilities and cognitive measures were controlled for age, sex, and education. Also, FDR correction for multiple comparisons across cognitive measures was performed using the Benjamini–Hochberg procedure per validation strategy, classification task, and center. This approach isolates disease-related cognitive signatures from demographic effects, allowing us to test whether EEG-derived predictions capture pathology-specific patterns of impairment rather than correlations driven by demographic structure (Figure 4).

For CN vs AD, predicted AD probability showed robust and domain-consistent associations across centers and validation tiers (Figure 4, Columns 1, 6 and 9). Higher AD probability was associated with poorer global cognition (MMSE), reduced semantic fluency, lower working-memory capacity (Digit Span forward and backward), impaired confrontation naming (BNT), and slower processing speed (Stroop time) and executive functions (TMT-A and TMT-B). Most of these associations remained significant after FDR correction and were preserved in multi- and cross-center validations. These findings indicate that EEG-derived scores tracked the severity of cognitive impairment characteristic of AD, rather than reflecting site-specific or age-related effects.

In CN vs MCI, correlations did not survive FDR correction and the pattern was fragmented across centers (Figure 4, Columns 2, 7 and 10). Although several analyses revealed negative associations with memory and fluency, consistent with expected prodromal deficits, these effects were not consistently replicated. This suggests that EEG–cognition coupling in MCI is weaker and more variable across populations, reflecting the heterogeneous and transitional nature of this stage, variability in diagnostic criteria, and its partial overlap with normal aging processes.

The MCI vs AD contrast showed significant negative correlations—albeit smaller than those for CN vs AD—with MMSE, semantic fluency, Digit Span forward, and BNT (Figure 4, Column 4). Considered together, the CN vs AD, CN vs MCI, and MCI vs AD analyses delineate a coherent gradient in which CN < MCI < AD, with MCI positioned closer to CN than to AD in terms of brain–behavior coupling.

The CN vs FTD distinction, available only at *BRAINLAT*_*cl*2_ with a small sample size, did not yield robust significant correlations after FDR correction (Figure 4, Column 3), limiting interpretation of this single-center contrast. Similarly, the FTD vs AD comparison showed non-significant associations (Figure 4, Columns 5, 8 and 11). However, these findings remain exploratory given the limited FTD sample size.

Overall, the distribution of associations outlines a graded continuum consistent with cognitive proximity between conditions. The density and stability of brain–behavior links were highest for CN vs AD, decreased for CN vs MCI, and became non-significant for executive domains in FTD, paralleling the performance gradient observed in Figure 2. This convergence indicates that the EEG classifiers organize individuals according to meaningful neurocognitive structure rather than purely statistical separations.

### 3.4 Interpretable EEG markers reveal disease-specific neurophysiological signatures

**Figure 5:**
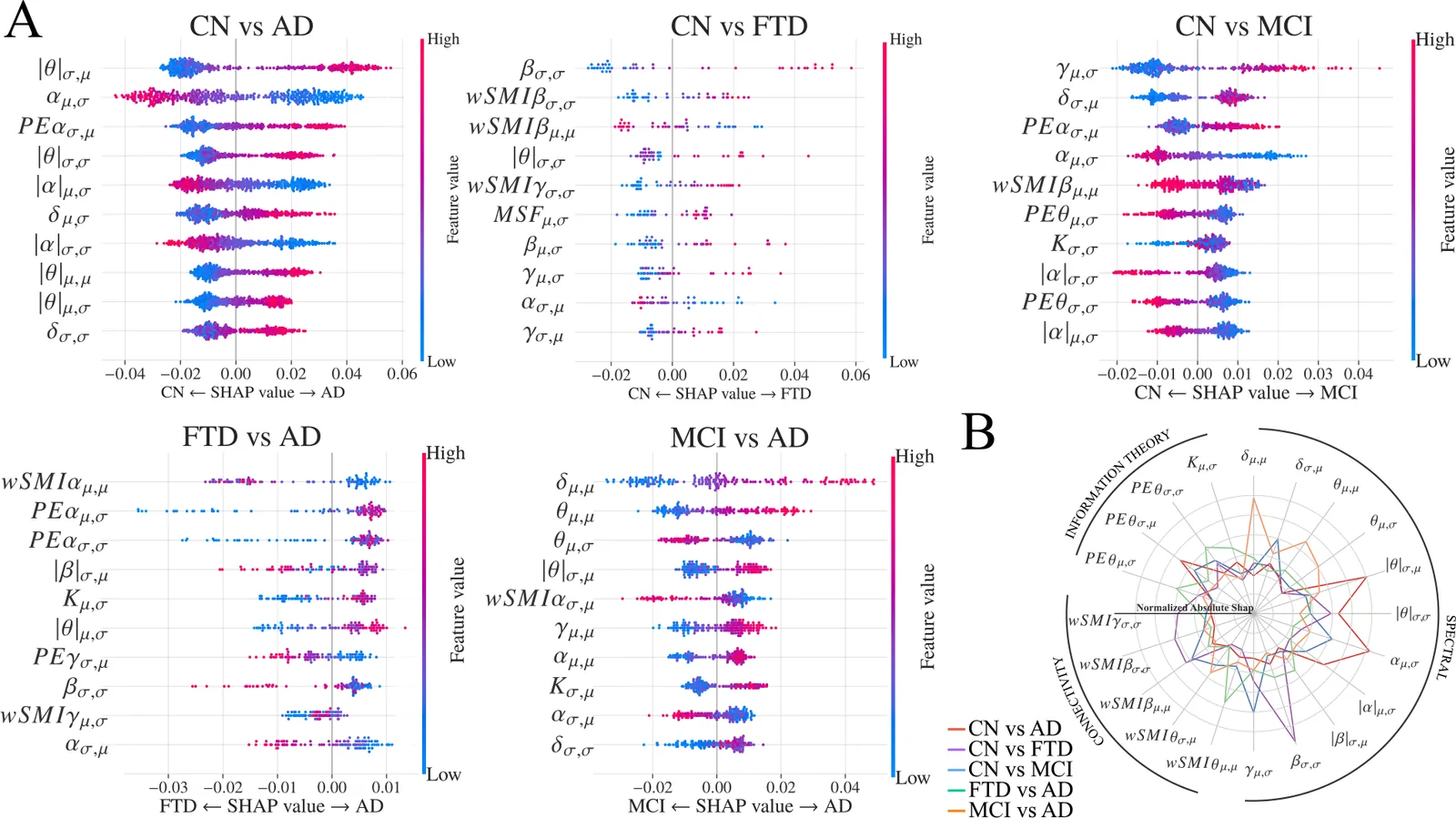
Feature importance analysis using SHAP values aggregated across all validation schemes. (A) Beeswarm plots displaying the top 10 most influential EEG features for each binary classification task. Each point represents a single prediction, with horizontal position indicating SHAP value (contribution to model output) and color reflecting feature value (blue: low, red: high). Features are ordered by mean rank of absolute SHAP values computed across all samples, pooling predictions from all validation schemes, datasets and repetitions. The notation indicates feature type and summarization method: Complexity, information theory, connectivity, and spectral markers, combined with summary statistics across channels (*μ*: mean, *σ*: standard deviation). (B) Polar plot summarizing normalized absolute SHAP values for features appearing in the top 5 of any classification task. Each axis represents a different EEG marker, with distance from the center indicating importance magnitude. Color-coded lines correspond to specific diagnostic contrasts, revealing task-specific feature utilization patterns.

SHAP values represent feature contributions to model output, with positive values pushing predictions toward the second diagnostic class and negative values toward the first class. In beeswarm plots, red points indicate high feature values and blue points indicate low values, with horizontal position showing SHAP contribution magnitude and direction (Figure 5A).

The most influential features for distinguishing CN vs AD patients were characterized by two distinct, opposing neurophysiological dynamics: a global reduction in alpha-band markers and a concurrent elevation in slow-wave (theta and delta) features (Figure 5A, leftmost panel). The degradation of alpha-band activity represented a primary discriminative signature, led by the temporal variability of alpha-band power (*α*_*μ,σ*_). For all alpha-related markers in the top features—including spatial variability of alpha-band permutation entropy (*PEα*_*σ,μ*_) and normalized alpha power variability (|*α*|_*μ,σ*_, |*α*|_*σ,σ*_)—lower feature values consistently pushed predictions toward AD (positive SHAP). This convergent pattern indicated a robust reduction in both temporal and spatial signal variability within the alpha band in pathological states.

Conversely, slow-wave markers exhibited the opposite directionality, reflecting pathological cortical slowing. The spatial variability of normalized theta power (|*θ*|_*σ,μ*_) ranked as the most influential feature overall, matching the discriminative magnitude of alpha temporal variability. For this and all other slow-wave features among the top ten—including theta variability (|*θ*|_*σ,σ*_, |*θ*|_*μ,σ*_), average normalized theta power (|*θ*|_*μ,μ*_), and delta variability (*δ*_*μ,σ*_, *δ*_*σ,σ*_)—higher values consistently drove predictions toward AD. Together, these results demonstrated that the classification between CN and AD relied on a complementary interplay between elevated low-frequency activity (theta and delta) and concurrent alpha-band degradation across multiple metrics, including power, entropy, and their spatiotemporal variability (Figure 5B).

The discrimination of FTD provided a sharp counterpoint to the AD signature, relying primarily on higher-frequency alterations (Figure 5A, second panel). Reduced spatiotemporal variability of beta-band power (*β*_*σ,σ*_) and connectivity (*wSMIβ*_*σ,σ*_, *wSMIβ*_*μ,μ*_), alongside gamma-band markers, represented the primary discriminative features. This dominance of beta and gamma alterations contrasted clearly with the alpha-theta pattern observed in AD (Figure 5B).

Detection of prodromal decline (CN vs MCI) revealed a more heterogeneous and attenuated marker profile (Figure 5A, third panel). The discrimination relied on a mixed pattern led by reduced gamma-band variability (*γ*_*μ,σ*_), accompanied by moderate changes in delta power (*δ*_*σ,μ*_) and alpha-band permutation entropy (*PEα*_*σ,μ*_). Consistent with the modest classification performance, SHAP distributions for MCI showed compressed magnitude ranges and increased overlap compared to established dementia.

Finally, direct contrasts between patient groups further highlighted these distinct physiological profiles (Figure 5A, fourth and fifth panels). FTD vs AD differentiation hinged primarily on connectivity and complexity measures, specifically average alpha connectivity (*wSMIα*_*μ,μ*_) and permutation entropy variability (*PEα*_*μ,σ*_). However, SHAP distributions for this contrast showed substantial overlap, reflecting its limited discriminative capacity. Conversely, the transition from MCI to AD was characterized primarily by progressive alterations in lower-frequency markers, including elevated theta and delta power (*θ*_*μ,μ*_, *δ*_*μ,μ*_) and reduced alpha connectivity (*wSMIα*_*σ,μ*_).

## 4 Discussion

This study aimed to evaluate whether automated multifeature EEG-based machine learning can provide clinically meaningful, generalizable, and interpretable biomarkers for dementia across heterogeneous international cohorts. Using a fully standardized pipeline applied to five independent centers, we demonstrated that EEG classifiers achieved robust and statistically significant discrimination between CN and AD across within-center, multi-center, and leave-one-center-out validation frameworks, confirming strong external generalizability. In contrast, classification performance was progressively reduced for earlier or more heterogeneous disease stages, including MCI and differential diagnosis between dementia syndromes (AD and FTD), reflecting increasing neurophysiological overlap. Model-derived probabilistic predictions at subject-level remained stable across validation regimes and showed significant associations with cognitive impairment independent of demographic factors, supporting their clinical validity. Interpretable feature analyses revealed biologically coherent neurophysiological signatures, including alpha-band degradation and increased slow-wave activity in AD, confirming mechanistic plausibility. Together, these findings support automated EEG-based classification as a relevant approach capable of providing partially robust, clinically meaningful, and biologically interpretable biomarkers, demonstrating substantial generalizability across diverse acquisition systems and populations, and highlighting EEG as a scalable and accessible modality for dementia detection.

We captured stable neurophysiological signatures of established neurodegeneration that transfer reliably across independent centers when assessing CN vs AD, extending previous single-site and limited multi-site studies that reported promising accuracy but uncertain external validity (Akbar et al., 2025; Yuan & Zhao, 2025; Peh et al., 2021; Ohal & Mantri, 2025). This robust performance likely stems from the fact that AD represents a well-defined neurobiological entity characterized by distinct and widespread electrophysiological alterations (Babiloni et al., 2021). The preservation of classification performance under leave-one-center-out validation suggests that the extracted markers reflect disease-specific neural alterations rather than center-specific artifacts, addressing a major limitation in prior EEG machine learning research. Similarly, well calibrated, single-patient probability estimates rather than simple binary classifications, enabling clinicians to assess diagnostic confidence and guide treatment planning accordingly (Van Calster et al., 2019). Calibration results confirmed that high-performing models, particularly CN vs AD across all validation strategies, exhibited excellent baseline calibration, indicating that raw classifier outputs already approximated well-calibrated probabilities.

The reduced performance observed for MCI aligns with the known biological and clinical heterogeneity of prodromal states, where neural alterations are subtler, more variable, and partially overlap with normal aging trajectories (Saini et al., 2025; Peng et al., 2026). Such comparisons seem to be particularly vulnerable to performance degradation under stringent cross-site validation (Jiao et al., 2023; Poil et al., 2013). Similarly, the moderate performance in MCI versus AD aligns with prior reports (Farina et al., 2020), reinforcing the view of MCI not as a monolithic category but as a broad severity spectrum (Huo et al., 2025). This spectrum encompasses a range from early stages that physiologically resemble healthy aging to late-stage presentations bordering to dementia. Such heterogeneity is further compounded by diverging clinical trajectories: while a subset of these patients will eventually convert to AD, others may remain clinically stable or never progress to dementia, leading to overlapping neurophysiological signatures that complicate binary classification (Petersen et al., 2014).

Similarly, the limited discrimination between dementia subtypes (AD vs FTD) likely reflects both overlapping pathophysiological mechanisms (Ibáñez et al., 2025; Caviedes et al., 2026) and the global summary feature representation used here, which prioritizes scalability over spatial specificity. This discrepancy with prior reports (Moguilner et al., 2024; Prado, Mejía, et al., 2023) could suggest a trade-off between framework scalability and spatial resolution. By reducing scalp data to global averages, our pipeline effectively captures the widespread, randomized neural activity characteristic of AD (Babiloni et al., 2021; Ahn et al., 2025). However, this global approach likely obscures the focal, topographically restricted markers required to distinguish FTD from the posterior-dominant pathology of AD (Nishida et al., 2011; Rostamikia et al., 2024). Collectively, these findings suggest limited discrimination between dementia subtypes.

The identification of alpha slowing and increased low-frequency activity as primary discriminative features is consistent with mechanistic models of AD (Coronel-Oliveros et al., 2024), reinforcing the biological validity of the classifier. This pattern directly reflects the hallmark cortical slowing of AD (Babiloni et al., 2021; Dauwels et al., 2011), a finding further supported by the complementary elevation of delta and theta variability. In contrast, prodromal MCI revealed an intermediate profile uniquely distinguished by gamma-band variability, capturing predementia heterogeneity (Jelic et al., 2000). Discrimination of FTD provided a sharp counterpoint to AD, relying on beta and gamma markers rather than alpha slowing (Nishida et al., 2011), while FTD-AD differentiation hinged on complexity measures consistent with divergent entropic signatures (Ahn et al., 2025). Thus, these results provide a rich neurophysiological characterization of the different diseases.

The association with cognitive impairment provides clinically meaningful disease characterization (Moguilner et al., 2024; Prado, Mejía, et al., 2023) and confirms that greater cognitive impairment is linked to higher disease probability estimates (Arevalo-Rodriguez et al., 2015; Ferrante et al., 2024; Huntley & Howard, 2010). Furthermore, predictions demonstrated expected demographic patterns, with higher AD probabilities in older individuals and lower probabilities in more educated participants, consistent with education’s well-established protective role against cognitive decline (Sharp & Gatz, 2011). As the associations were preserved after controlling for age, sex, and education, EEG-derived predictions reflect pathological processes and are unlikely to be mainly driven by confounds. Collectively, these results support the view that multifeatured EEG markers can provide robust, mechanistically grounded indicators of neurodegeneration, while highlighting that prodromal and differential diagnosis remain inherently more challenging due to biological heterogeneity.

This study has several key strengths. The multi-center design spanning diverse acquisition systems, populations, and geographic regions provides unusually strong evidence of external validity compared with prior single-site or limited validation studies. The hierarchical validation strategy, including leave-one-center-out evaluation, represents a rigorous test of true generalizability rarely implemented in EEG dementia research. The use of a fully automated and standardized preprocessing and feature extraction pipeline minimizes operator-dependent variability and enhances reproducibility and scalability across clinical environments. The integration of probability calibration and cognitive association analyses demonstrates direct clinical relevance beyond binary classification, supporting the potential utility of EEG-derived predictions as quantitative biomarkers. The interpretability analysis provides mechanistic transparency by linking classifier outputs to known neurophysiological signatures of neurodegeneration. Finally, the feature representation was designed to remain compatible with heterogeneous and low-density EEG systems, enhancing feasibility for deployment in real-world and resource-limited clinical settings.

Several limitations should be considered. Sample size imbalance across diagnostic contrasts, particularly for frontotemporal dementia and certain cross-center comparisons, limited statistical power and constrained differential diagnosis evaluation. The use of spatially aggregated features, while improving robustness and scalability, likely reduced sensitivity to focal or topographically specific alterations relevant for distinguishing dementia subtypes. The conservative pipeline intentionally avoided hyperparameter tuning to minimize overfitting risk and ensure transferability to unseen populations; a trade-off that enhanced confidence in external validity at the cost of absolute performance metrics. Nevertheless, hyperparameter optimization could be applied in clinical deployment settings where maximum performance is desired. The cross-sectional design precludes direct assessment of longitudinal progression and predictive value for disease conversion, particularly for mild cognitive impairment. Diagnostic classification relied on clinical criteria without systematic or molecular biomarker confirmation (ATN Framework, Caviedes et al., 2026), which may introduce heterogeneity. Although the standardized pipeline minimizes variability, residual differences in acquisition hardware, preprocessing, and cohort characteristics may still influence performance. Finally, while the classifier demonstrated strong generalizability for established Alzheimer’s disease, performance for early detection and differential diagnosis remains limited, highlighting the need for multimodal integration and larger multicenter datasets. Future studies could leverage federated learning approaches which offer a promising avenue to address these limitations by enabling distributed model training across institutions while preserving patient privacy, achieving necessary sample sizes without data centralization (Peh et al., 2021).

In conclusion, this study demonstrates that automated multifeature EEG-based machine learning can achieve clinically meaningful and externally generalizable detection of Alzheimer’s disease across heterogeneous international cohorts while providing calibrated, biologically interpretable probability estimates linked to cognitive impairment. These findings establish EEG as a robust and scalable functional biomarker capable of supporting dementia screening across diverse clinical environments. More broadly, the results highlight the importance of rigorous cross-center validation, interpretable modeling, and clinically grounded evaluation for translating machine learning biomarkers into real-world applications. Future work integrating longitudinal data, multimodal biomarkers, and larger multicenter cohorts will further enable EEG-based precision diagnostics and scalable global dementia care.

## Supplementary Material

Supplement 1

## Figures and Tables

**Table 1:** Demographic of participants across the five contributing centers. The table reports the number of participants (*n*), sex distribution (female:male), mean age with standard deviation (years), and mean years of education with standard deviation for each diagnostic group: cognitively normal (CN), mild cognitive impairment (MCI), Alzheimer’s disease (AD), and frontotemporal dementia (FTD). The bottom rows summarize the total distribution across all centers.

Dataset	Variable	CN	MCI	AD	FTD

	n	0	0	26	24
*BRAINLAT*_*ar*_	Sex (female:male)	—	—	17:9	9:15
	Age (years)	—	—	74.50 (7.50)	69.75 (11.07)
	Years of education	—	—	9.23 (4.88)	14.21 (4.47)
	n	27	0	19	0
*BRAINLAT*_*cl*1_	Sex (female:male)	15:12	—	10:9	—
	Age (years)	73.93 (4.21)	—	77.89 (9.76)	—
	Years of education	13.41 (4.50)	—	11.32 (4.99)	—
	n	30	0	38	16
*BRAINLAT*_*cl*2_	Sex (female:male)	5:25	—	23:15	5:11
	Age (years)	63.30 (10.25)	—	69.92 (7.24)	71.38 (5.50)
	Years of education	16.67 (3.48)	—	13.20 (4.70)	14.33 (3.42)
	n	98	100	97	0
*IUEFM*_*tr*_	Sex (female:male)	63:35	47:53	64:33	—
	Age (years)	65.63 (8.33)	73.62 (6.63)	74.28 (5.12)	—
	Years of education	12.72 (4.45)	10.34 (4.64)	8.69 (4.44)	—
	n	66	54	0	0
*USCO – UCC*_*co*_	Sex (female:male)	53:12	46:8	—	—
	Age (years)	60.29 (6.25)	63.54 (7.01)	—	—
	Years of education	14.08 (5.20)	7.43 (4.69)	—	—
	n	91	0	78	0
*USP*_*br*_	Sex (female:male)	56:35	—	50:21	—
	Age (years)	60.63 (16.77)	—	80.94 (8.35)	—
	Years of education	12.40 (3.47)	—	10.65 (4.18)	—

	n	312	154	258	40
**Total**	Sex (female:male)	192:119	93:61	164:87	14:26
	Age (years)	63.55 (11.76)	70.08 (8.29)	75.80 (8.00)	70.40 (9.20)
	Years of education	13.35 (4.44)	9.29 (4.85)	10.16 (4.72)	14.26 (4.05)

**Table 2:** Classification performance across diagnostic contrasts, centers, and validation strategies. For each comparison, performance metrics are computed across 500 repeated runs of the full evaluation pipeline and summarized as *μ* ± *σ*. Reported metrics include discrimination (area under the ROC curve, AUC), precision (Prec), sensitivity (Sens), and confusion matrix components (true negatives [TN], false positives [FP], false negatives [FN], true positives [TP]). The reported variability reflects robustness to dataset split differences and model stochasticity. In the Cross-center (Cc) validation, where train/test splits are fixed, this variability arises exclusively from model stochasticity. Effect size is quantified as the difference between the mean AUC obtained with true labels and the mean AUC obtained under permuted labels (ΔAUC). Statistical significance is assessed using a permutation-based approach, where *p*_*val*_ corresponds to the empirical probability that the AUC obtained under permuted labels exceeds the mean AUC obtained with true labels, providing a one-sided test of performance above chance level. Calibration metrics include *Cal*_Δ_ (reduction in mean squared error after Platt scaling calibration) and *Cal*_*err*_ (residual mean squared error between non-calibrated probabilities and observed outcomes). Three validation strategies were employed: within-center (Wc), multi-center (Mc), and cross-center (Cc).

Contrast	Center	Val	AUC	Prec	Sens	ΔAUC	TN	FP	FN	TP	*p_val_*	*Cal*_Δ_	*Cal*_*err*_

CN vs AD	*BRAINLAT*_*cl*1_	Cc	**0.72**±0.03	**0.88**±0.11	**0.21**±0.04	**0.21**±0.09	**26.42**±0.52	**0.58**±0.52	**15.05**±0.68	**3.95**±0.68	0.01	0.00	0.04
		Mc	**0.73**±0.03	**0.71**±0.11	**0.36**±0.06	**0.27**±0.11	**24.03**±1.36	**2.97**±1.36	**12.10**±1.23	**6.90**±1.23	0.01	0.00	0.02
		Wc	**0.74**±0.05	**0.63**±0.07	**0.54**±0.09	**0.27**±0.12	**20.96**±1.49	**6.04**±1.49	**8.82**±1.63	**10.18**±1.63	0.02	0.00	0.00
	
	*IUEFM*_*tr*_	Cc	**0.78**±0.01	**0.74**±0.03	**0.64**±0.03	**0.27**±0.04	**76.40**±3.42	**21.60**±3.42	**34.91**±2.53	**62.09**±2.53	0.00	0.00	0.01
		Mc	**0.83**±0.01	**0.80**±0.03	**0.68**±0.02	**0.34**±0.05	**81.74**±2.64	**16.26**±2.64	**31.18**±2.31	**65.82**±2.31	0.00	0.00	0.01
		Wc	**0.83**±0.01	**0.76**±0.02	**0.73**±0.02	**0.34**±0.05	**75.58**±2.91	**22.42**±2.91	**25.84**±2.30	**71.16**±2.30	0.00	0.00	0.01
	
	*BRAINLAT*_*cl*2_	Cc	**0.82**±0.02	**0.70**±0.02	**0.88**±0.03	**0.32**±0.07	**15.84**±1.26	**14.16**±1.26	**4.45**±0.97	**33.55**±0.97	0.00	0.00	0.01
		Mc	**0.79**±0.02	**0.70**±0.02	**0.84**±0.03	**0.31**±0.08	**16.45**±1.09	**13.55**±1.09	**5.94**±1.20	**32.06**±1.20	0.00	0.00	0.01
		Wc	**0.76**±0.03	**0.71**±0.02	**0.81**±0.03	**0.30**±0.11	**17.61**±0.98	**12.39**±0.98	**7.20**± 1.31	**30.80**±1.31	0.00	0.00	0.00
	
	*USP*_*br*_	Cc	**0.79**±0.01	**0.57**±0.01	**0.86**±0.02	**0.29**±0.04	**39.68**±2.41	**51.32**±2.41	**10.63**±1.83	**67.37**±1.83	0.00	0.00	0.03
		Mc	**0.79**±0.01	**0.66**±0.02	**0.75**±0.03	**0.30**±0.06	**60.52**±2.53	**30.48**±2.53	**19.80**±1.96	**58.20**±1.96	0.00	0.00	0.01
		Wc	**0.77**±0.01	**0.68**±0.03	**0.63**±0.04	**0.28**±0.06	**68.39**±2.40	**22.61**±2.40	**29.09**±2.78	**48.91**±2.78	0.00	0.00	0.00

CN vs FTD	*BRAINLAT*_*cl*2_	Wc	**0.63**±0.05	**0.37**±0.10	**0.20**±0.07	**0.17**±0.13	**24.43**±1.47	**5.57**±1.47	**12.77**±1.06	**3.23**±1.06	0.12	0.00	0.02

CN vs MCI	*IUEFM*_*tr*_	Cc	**0.57**±0.03	**0.52**±0.02	**0.80**±0.07	**0.07**±0.05	**25.33**±9.25	**72.67**±9.25	**20.08**±7.48	**79.92**±7.48	0.11	0.03	0.03
		Mc	**0.69**±0.02	**0.64**±0.02	**0.62**±0.03	**0.20**±0.06	**62.30**±2.88	**35.70**±2.88	**37.54**±2.92	**62.46**±2.92	0.00	0.00	0.03
		Wc	**0.68**±0.02	**0.62**±0.02	**0.63**±0.03	**0.20**±0.06	**59.78**±2.72	**38.22**±2.72	**37.09**±2.70	**62.91**±2.70	0.00	0.00	0.01
	
	*USCO – UCC_co_*	Cc	**0.52**±0.02	**0.46**±0.02	**0.73**±0.04	**0.02**±0.06	**19.46**±3.85	**46.54**±3.85	**14.35**±2.25	**39.65**±2.25	0.42	0.00	0.12
		Mc	**0.53**±0.03	**0.48**±0.04	**0.38**±0.04	**0.05**±0.07	**43.70**±2.78	**22.30**±2.78	**33.75**±2.27	**20.25**±2.27	0.37	0.00	0.03
		Wc	**0.52**±0.03	**0.46**±0.04	**0.38**±0.05	**0.04**±0.08	**41.79**±3.12	**24.21**±3.12	**33.43**±2.60	**20.57**±2.60	0.40	0.00	0.06

FTD vs AD	*BRAINLAT*_*ar*_	Cc	**0.48**±0.03	**0.51**±0.04	**0.53**±0.08	**−0.01**±0.09	**10.73**±2.15	**13.27**±2.15	**12.28**±1.98	**13.72**±1.98	0.61	0.01	0.11
		Mc	**0.44**±0.06	**0.50**±0.04	**0.61**±0.08	**−0.03**±0.12	**8.00**±1.82	**16.00**±1.82	**10.21**±2.04	**15.79**±2.04	0.61	0.01	0.18
		Wc	**0.42**±0.06	**0.47**±0.06	**0.47**±0.09	**−0.03**±0.12	**10.35**±1.99	**13.65**±1.99	**13.66**±2.24	**12.34**±2.24	0.61	0.01	0.17
	
	*BRAINLAT*_*cl*2_	Cc	**0.44**±0.03	**0.68**±0.03	**0.41**±0.05	**−0.05**±0.09	**8.79**±0.93	**7.21**±0.93	**22.56**±1.74	**15.44**±1.74	0.76	0.03	0.16
		Mc	**0.56**±0.05	**0.70**±0.02	**0.84**±0.04	**0.10**±0.12	**2.21**±0.92	**13.79**±0.92	**6.19**±1.35	**31.81**±1.35	0.26	0.01	0.13
		Wc	**0.54**±0.05	**0.69**±0.02	**0.86**±0.04	**0.08**±0.12	**1.58**±0.98	**14.42**±0.98	**5.47**±1.59	**32.53**±1.59	0.36	0.00	0.17

MCI vs AD	*IUEFM*_*tr*_	Wc	**0.60**±0.02	**0.57**±0.03	**0.51**±0.03	**0.11**±0.06	**63.31**±3.45	**36.69**±3.45	**47.47**±2.69	**49.53**±2.69	0.04	0.01	0.05

## Data Availability

The derived markers generated and analyzed in this study are publicly available in a Zenodo repository (Belloli, 2026a). These data include all computed features used for statistical analyses and model training, enabling full reproducibility of the reported results. The raw data are not publicly available due to ethical and legal restrictions related to sensitive clinical information and because they are owned by third-party institutions. Researchers interested in accessing the raw data should contact the original data providers directly and comply with their respective data access procedures and regulatory requirements.

## 6 Code Availability

All feature extraction procedures, classification pipelines, and statistical analyses required to reproduce the results reported in this study are publicly available in a Zenodo repository (Belloli, 2026b). The repository includes the full source code, a reproducible Python environment (Conda) with all required dependencies specified, and a detailed README providing step-by-step instructions for reproducing the experimental pipeline and analyses.
