# Supplementary material for: Generalizability of EEG-Based DEMENTIA Classifiers: A Multicenter study of Alzheimer’s, MCI, and FTD
Source: medRxiv. 2026 Jul 29:2026.07.28.26359133. Preprint. [Version 1] doi: 10.64898/2026.07.28.26359133 (PMC13484356; doi:10.64898/2026.07.28.26359133)
Supplement: Supplementary file 1 [file NIHPP2026.07.28.26359133v1-supplement-1.pdf]

medRxiv preprint doi: https://doi.org/10.64898/2026.07.28.26359133; this version posted July 29, 2026. The copyright holder for this preprint (which was not certified by peer review) is the author/funder, who has granted medRxiv a license to display the preprint in perpetuity.
It is made available under a CC-BY 4.0 International license .

10 Supplemental material

10.1 Robust cross, multi-center generalization are maintained after down-sampling for centers and class balance

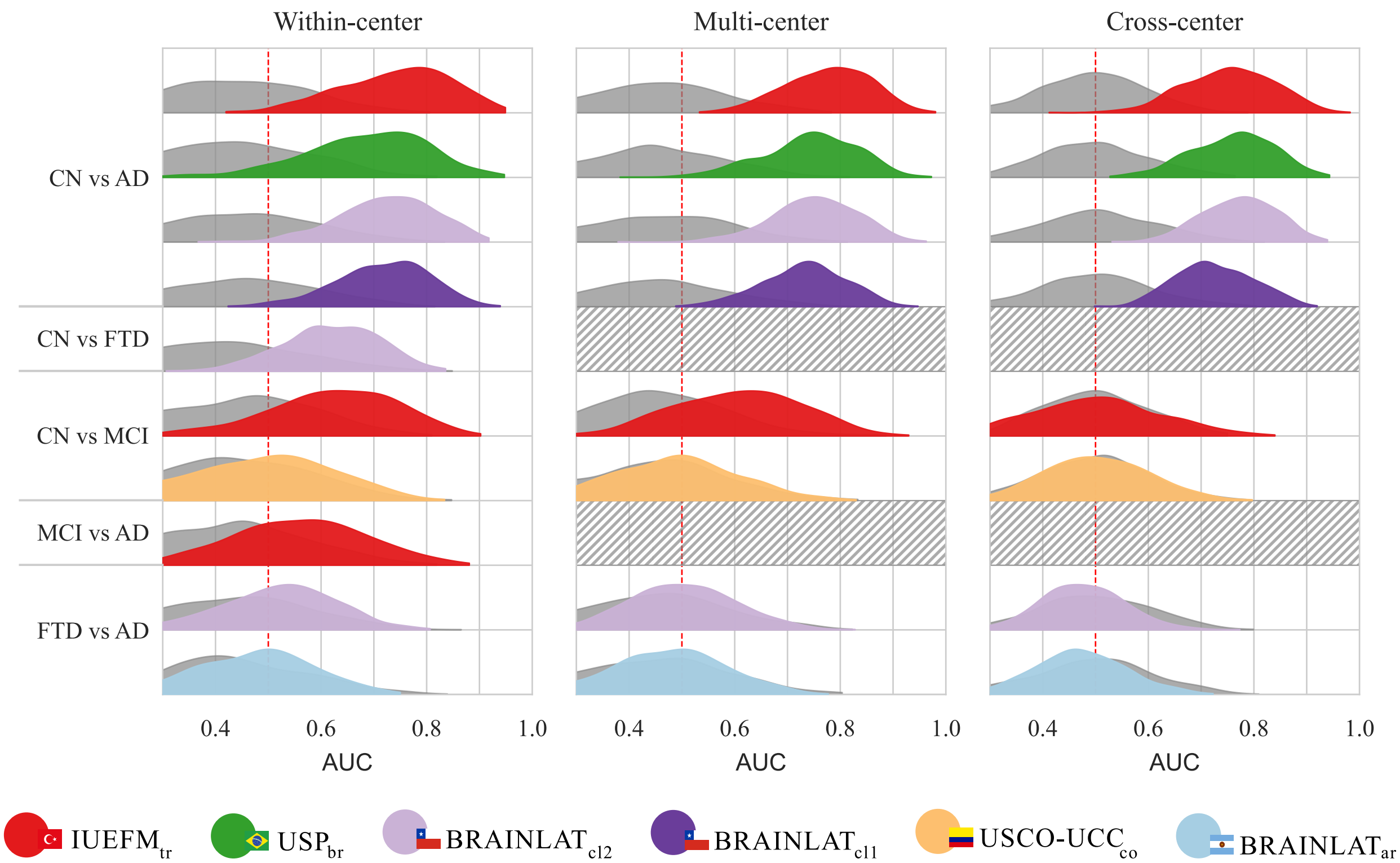

Figure S1: **Classification performance under downsampled balanced conditions across validation strategies and diagnostic comparisons** (16 **samples per center and diagnostic).** Each panel represents a different validation approach: *Within-center* (left) shows performance using Stratified by diagnostic category K-Fold cross-validation within individual centers; *Multi-center* (middle) displays results from pooled data across multiple sites using Stratified K-Fold cross-validation; *Cross-center* (right) illustrates generalization performance using Leave-One-Center-Out cross-validation, where each center was iteratively held out as an independent test set. Prior to model training, a repeated stratified downsampling procedure was applied to construct balanced datasets with equal sample sizes across all combinations of diagnostic category and center. Distribution plots show the AUC scores across 500 repetitions of the full pipeline (downsampling + cross-/leave-one-center-out- validations) for each binary classification tasks (y-axis): CN vs AD, CN vs FTD, CN vs MCI, MCI vs AD, and FTD vs AD. Colors correspond to different acquisition centers/datasets: $BRAINLAT_{ar}$ (light blue), $BRAINLAT_{cl1}$ (dark purple), $BRAINLAT_{cl2}$ (purple), $IUEFM_{tr}$ (red), $USCO-UCC_{co}$ (orange), and USP_{br} (green). Hatched areas indicate diagnostic comparisons not available at particular centers due to absence of required diagnostic categories. The red dashed line at AUC = 0.5 marks chance-level performance. Overall, performance patterns closely mirror those observed in the full dataset analysis, with slightly increased variability and modest reductions in AUC consistent with reduced sample sizes.

To evaluate the extent to which the results reported in the main analysis could be influenced by imbalances in sample size across diagnostic categories and acquisition centers, we performed a repeated stratified downsampling procedure prior to model training and evaluation. For each repetition, we constructed a new balanced dataset by randomly sampling, without replacement, an equal number of observations from each combination of diagnostic category and center. The number of samples per subgroup was fixed at 16 across all centers and classes and determined by the smallest available subgroup to ensure feasibility. This procedure resulted in datasets with identical class and center distributions across repetitions, thereby removing potential confounding effects arising from unequal sample sizes.

For each resampled dataset, the same validation strategies described in the main analysis (See section 2.4) were applied using identical model configurations and evaluation pipelines.

medRxiv preprint doi: https://doi.org/10.64898/2026.07.28.26359133; this version posted July 29, 2026. The copyright holder for this preprint (which was not certified by peer review) is the author/funder, who has granted medRxiv a license to display the preprint in perpetuity.
It is made available under a CC-BY 4.0 International license .

The results of this control analysis are presented in Supplementary Figure S1. Overall, the performance patterns were highly consistent with those observed in the main analysis without downsampling. In particular, the relative differences across centers, diagnostic comparisons, and validation strategies were preserved, supporting the robustness of the main findings.

Two expected differences emerged as a direct consequence of the downsampling procedure. First, variability across repetitions increased relative to the main analysis. This reflects the fact that each repetition is performed on a different subset of participants, whereas in the original analysis all repetitions are conducted on the same dataset, with variability arising solely from differences in cross-validation splits and model stochasticity. Second, a modest decrease in performance was observed across conditions. This reduction is expected given the smaller number of training samples available in each repetition, which limits model capacity. Importantly, this decrease was relatively small and not proportional to the magnitude of downsampling, despite some datasets being substantially reduced in size (e.g., from 78,91 or 100, to 16 samples), indicating that the learned patterns remain robust even under constrained sample sizes.

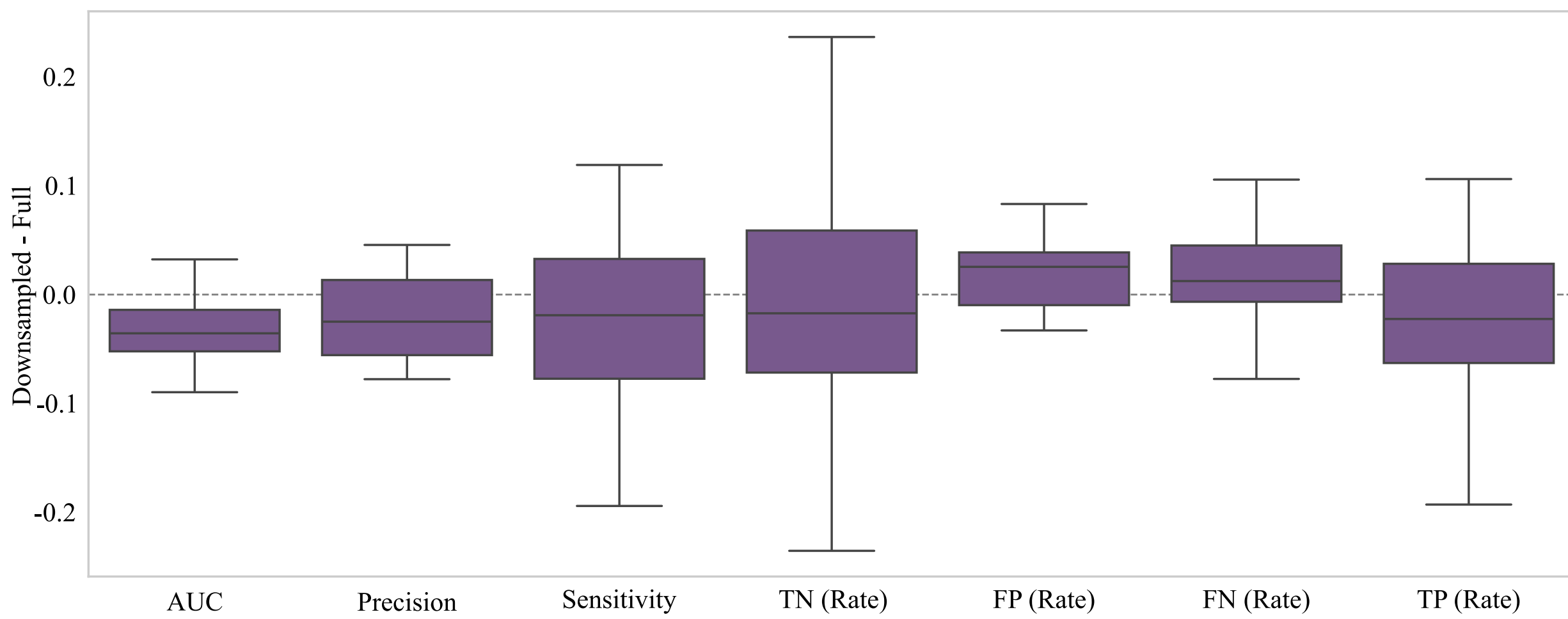

Figure S2: Comparison of performance metric distributions between the original and downsampled analyses across all centers, diagnostic contrasts, and validation strategies. For each metric (AUC, precision, sensitivity, and confusion matrix rates), distributions are shown for the full dataset and the downsampled dataset.

To further quantify the agreement between the original and downsampled analyses, we examined the paired differences in performance metrics across all centers, diagnostic comparisons, and validation strategies (Supplementary Figure S2). For each metric, we computed the difference between the downsampled and original results, such that values centered around zero indicate minimal impact of downsampling.

Across all evaluated metrics, the distributions of paired differences were just a small apart from zero (no differences). While some variability was observed—particularly for sensitivity and true negative rates—the overall magnitude of these deviations remained small. Importantly, no systematic degradation in performance was evident across metrics.

Taken together, the near-zero centering of the distributions and the limited spread of differences provide converging evidence that downsampling does not meaningfully alter model performance. These findings indicate that the performance patterns observed in the main analysis are not driven by imbalances in sample size across centers or diagnostic groups, but instead reflect robust and consistent signals that persist under controlled, balanced sampling conditions.

10.2 EEG-derived predictions align with cognitive impairment clinical tests

To evaluate whether model-predicted probabilities captured clinically meaningful patterns of cognitive and demographic variation, Spearman correlations were computed between predicted probabilities and available demographic and neuropsychological variables across all validation strategies and diagnostic comparisons. Model predictions ranged from 0 (indicating the first diagnostic class) to 1 (indicating the second class) in each binary comparison. Cognitive test scores were inverted when necessary to ensure consistent directionality, such that lower scores uniformly reflected greater impairment. Statistical significance was determined with FDR correction using the Benjamini–Hochberg procedure to account for multiple comparisons per validation strategy, classification contrast, and center.

medRxiv preprint doi: https://doi.org/10.64898/2026.07.28.26359133; this version posted July 29, 2026. The copyright holder for this preprint (which was not certified by peer review) is the author/funder, who has granted medRxiv a license to display the preprint in perpetuity.
It is made available under a CC-BY 4.0 International license .

10.2.1 CN vs AD Classification

Demographic variables showed selective associations with predicted AD probabilities across validation strategies (Figure S3, leftmost columns). Age demonstrated positive correlations at specific centers, with values ranging from 0.29 to 0.34 in within-center validation, 0.34 to 0.37 in multi-center validation, and 0.32 to 0.35 in cross-center validation, indicating that older individuals at these sites received higher AD probabilities independent of diagnostic group. Education showed minimal significant associations after FDR correction, with most correlations non-significant, suggesting that the primary discriminative features captured by EEG markers operated largely independently of educational attainment.

Cognitive performance revealed substantially stronger associations with predicted probabilities across all validation strategies. Global cognitive screening through Mini-Mental State Examination showed robust negative correlations, ranging from -0.25 to -0.54 within individual centers, -0.33 to -0.55 with multi-center training, and -0.30 to -0.49 when tested on unseen sites (Figure S3). These patterns indicated that individuals with lower cognitive function consistently received higher AD probabilities. Semantic fluency paralleled this pattern with significant negative correlations across validation strategies (r_{wc} = -0.28 to -0.51, r_{mc} = -0.34 to -0.50, r_{cc} = -0.22 to -0.50), suggesting that verbal fluency deficits characteristic of Alzheimer's disease corresponded reliably with EEG-based probability estimates.

Working memory capacity demonstrated differential sensitivity depending on task demands. Backward digit span showed stronger associations (r_{wc} = -0.25 to -0.46, r_{mc} = -0.38 to -0.48, r_{cc} = -0.39 to -0.41) than forward span (r_{wc} = -0.20 to -0.21, r_{mc} = -0.21 to -0.32, r_{cc} = -0.13 to -0.33), with this differential pattern suggesting particular sensitivity to executive control aspects of working memory. Confrontation naming through Boston Naming Test revealed strong negative correlations at $IUEFM_{tr}$, particularly evident in within-center (r_{wc} = -0.58), multi-center (r_{mc} = -0.56), and cross-center (r_{cc} = -0.48) validation, indicating that semantic memory impairment aligned consistently with EEG-derived probability estimates.

Processing speed and executive function showed positive relationships with disease probabilities, as completion times increase with cognitive decline (Figure S3). Trail Making Test A demonstrated significant associations (r_{wc} = 0.31-0.38, r_{mc} = 0.34-0.42, r_{cc} = 0.19-0.42), while Trail Making Test B showed similar patterns (r_{wc} = 0.25-0.28, r_{mc} = 0.35-0.44, r_{cc} = 0.26-0.46). These associations persisted across validation strategies, indicating that processing speed deficits corresponded reliably with predicted AD probabilities.

10.2.2 CN vs MCI Classification

The distinction between cognitively normal individuals and those with mild cognitive impairment revealed more variable and generally weaker cognitive-prediction associations (Figure S3, second columns). Demographic variables demonstrated minimal significant associations after FDR correction, with age showing small positive correlations at $IUEFM_{tr}$ (r_{wc} = 0.25, r_{mc} = 0.22) and education remaining non-significant across centers. Mini-Mental State Examination showed variable negative correlations across centers and validation strategies, ranging from -0.25 to -0.42 within centers, -0.33 to -0.49 with multi-center training, and -0.30 to -0.49 when tested on unseen sites.

Semantic fluency demonstrated negative correlations primarily at $IUEFM_{tr}$ across validation strategies (r_{wc} = -0.28, r_{mc} = -0.48, r_{cc} = -0.50), while working memory measures showed selective associations with digit span backward (r_{wc} = -0.25, r_{mc} = -0.38, r_{cc} = -0.39) at $IUEFM_{tr}$. Processing speed measures revealed patterns concentrated at specific centers (Figure S3). Trail Making Test A (r_{wc} = 0.38, r_{mc} = 0.42, r_{cc} = 0.42) and Test B (r_{wc} = 0.25, r_{mc} = 0.44, r_{cc} = 0.46) showed significant positive correlations at $IUEFM_{tr}$ across validation strategies, indicating that processing speed and executive function deficits showed correspondence with EEG-based predictions at specific centers.

10.2.3 Other Diagnostic Comparisons

The CN vs FTD distinction, available only at $BRAINLAT_{cl2}$, demonstrated moderate associations with age (r_{wc} = 0.29), Mini-Mental State Examination (r_{wc} = 0.26), and processing speed measures (Figure S3, third column of within-center panel). Interpretation remained limited by single-center availability. The MCI vs AD comparison revealed significant associations with Mini-Mental State Examination (r_{wc} = -0.15), semantic fluency (r_{wc} = -0.20), and Trail Making Test performance (r_{wc} = 0.22-0.27), aligning with the moderate discriminative performance observed for this diagnostic comparison.

The FTD vs AD differentiation revealed minimal significant associations with demographic or cognitive measures after FDR correction across validation strategies (rightmost columns, Figure S3). Most cognitive measures including Mini-Mental State Examination, semantic fluency, and digit span showed predominantly non-significant correlations. Trail Making Test demonstrated scattered significant associations at $BRAINLAT_{ar}$ (r_{wc} = 0.40-0.49, r_{mc} = 0.23-0.36,

medRxiv preprint doi: https://doi.org/10.64898/2026.07.28.26359133; this version posted July 29, 2026. The copyright holder for this preprint (which was not certified by peer review) is the author/funder, who has granted medRxiv a license to display the preprint in perpetuity.
It is made available under a CC-BY 4.0 International license .

r_{cc} = 0.80 for Test B), though patterns were inconsistent across sites. This sparse pattern corresponded with the limited discriminative capacity observed for this diagnostic comparison.

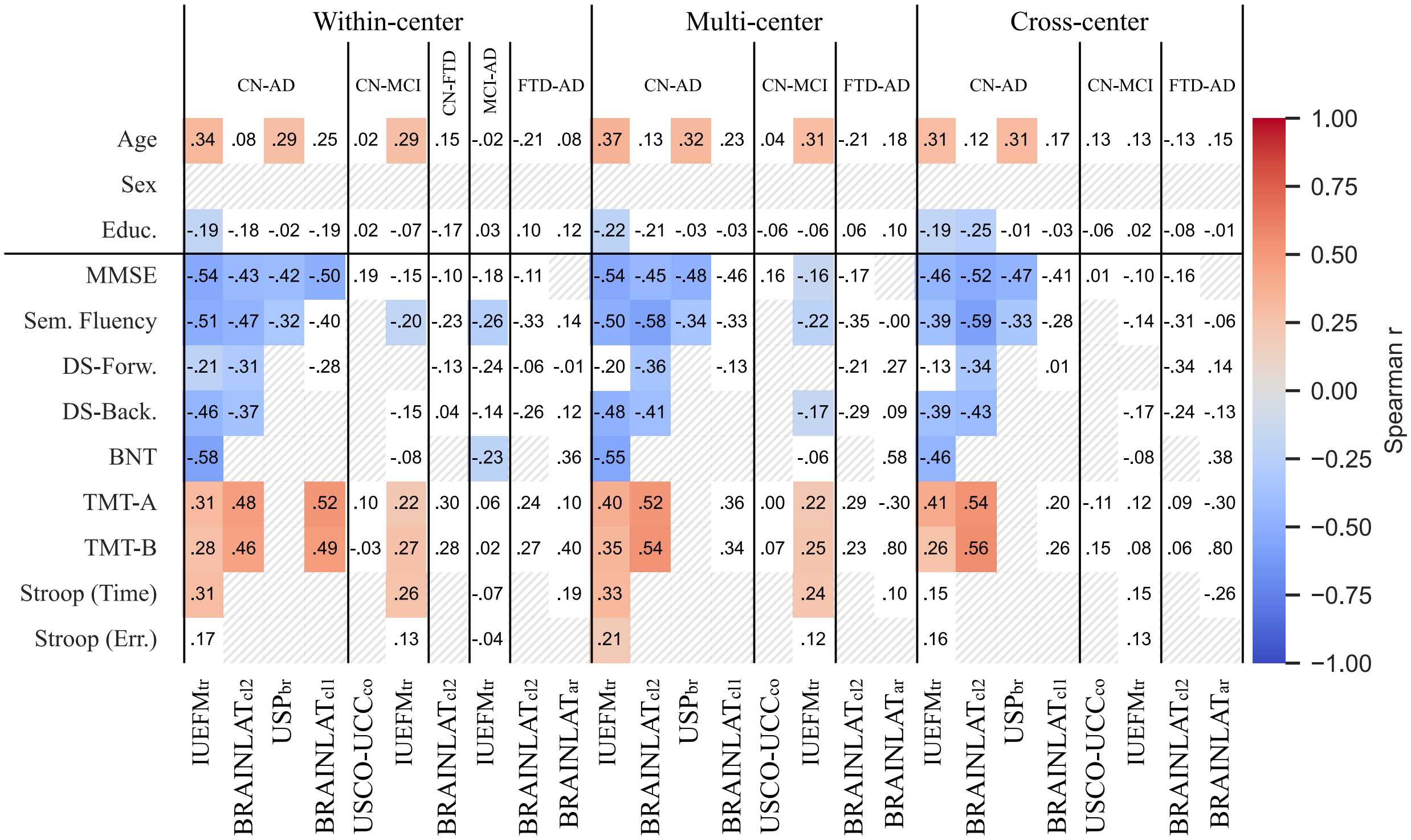

Figure S3: Spearman correlations between model-predicted probabilities and demographic/cognitive measures across validation schemes. Each column represents a specific classification task (CN-AD, CN-MCI, CN-FTD, MCI-FTD, FTD-AD) within three validation approaches: within-center (left panel), multi-center (middle panel), and cross-center (right panel). Model predictions ranged from 0 (first class) to 1 (second class) in each binary comparison. Cognitive test scores were inverted when necessary to ensure consistent directionality, with lower performance indicating greater impairment. Correlation coefficients are displayed within cells, with color intensity representing magnitude and direction (blue: negative, red: positive). Hatched cells indicate non-significant correlations after false discovery rate correction.

10.3 Feature importance stability across cross-validation repetitions

To assess whether the EEG features identified as most influential by SHAP analysis were consistently selected across repeated cross-validation runs, rather than being artefacts of particular train/test splits, we computed the Jaccard similarity of top-10 feature sets across all pairwise repetition pairs. For each repetition and each diagnostic contrast, the top 10 features were identified by ranking features according to their mean absolute SHAP value on the held-out test fold. The Jaccard index between each pair of repetitions was then computed as the cardinality of the intersection of the two feature sets divided by the cardinality of their union. This procedure was applied independently for each diagnostic contrast and validation strategy, yielding a distribution of pairwise Jaccard values whose mean and standard deviation quantify the stability and reproducibility of the identified feature signatures.

Results are presented in Supplementary material Table S1 and visualised in Supplementary material Figure S4. Feature importance stability mirrored the classification performance gradient described in the main analysis. For CN vs AD, Jaccard similarity was high across validations (0.77 to 0.89), indicating that the same subset of features (primarily alpha-band degradation and slow-wave increases) was reliably recovered regardless of the training configuration. This consistency supports the interpretation that the discriminative signature for established Alzheimer's disease is robust and not sensitive to dataset composition, reinforcing the biological plausibility of the identified biomarkers.

For CN vs MCI, feature stability was reduced across all validation strategies but maintained considerable stability (0.38 to 0.66). This stability range is consistent with the heterogeneous and attenuated neurophysiological alterations at the prodromal stage and echoes the modest and variable classification performance for this contrast. The particularly low mean Jaccard value for the Multi-center case suggests that the feature landscape shifts substantially when models are

medRxiv preprint doi: https://doi.org/10.64898/2026.07.28.26359133; this version posted July 29, 2026. The copyright holder for this preprint (which was not certified by peer review) is the author/funder, who has granted medRxiv a license to display the preprint in perpetuity.
It is made available under a CC-BY 4.0 International license .

trained on pooled heterogeneous data, likely because different centers capture different facets of early neurodegeneration in their MCI cohorts. A similar pattern of reduced stability was observed for FTD vs AD (0.33 to 0.53) and MCI vs AD (0.50), consistent with the limited and heterogeneous discriminative capacity observed for these contrasts in the main analysis. The CN vs FTD contrast, available only within $BRAINLAT_{cl2}$, showed intermediate stability (0.61).

Taken together, these findings demonstrate that SHAP-identified biomarkers are reproducible in proportion to the discriminability of the underlying contrast. High feature stability for CN vs AD across validation regimes provides additional evidence that the alpha-theta signature reported in the main analysis is a genuine, center-independent neurophysiological marker of established Alzheimer's disease. The graded reduction in stability for MCI-related and FTD contrasts further underscores the inherently more variable neural phenotypes at these diagnostic boundaries, and highlights the need for larger multicentric samples and more targeted feature representations to achieve reliable identification of prodromal and differential dementia biomarkers.

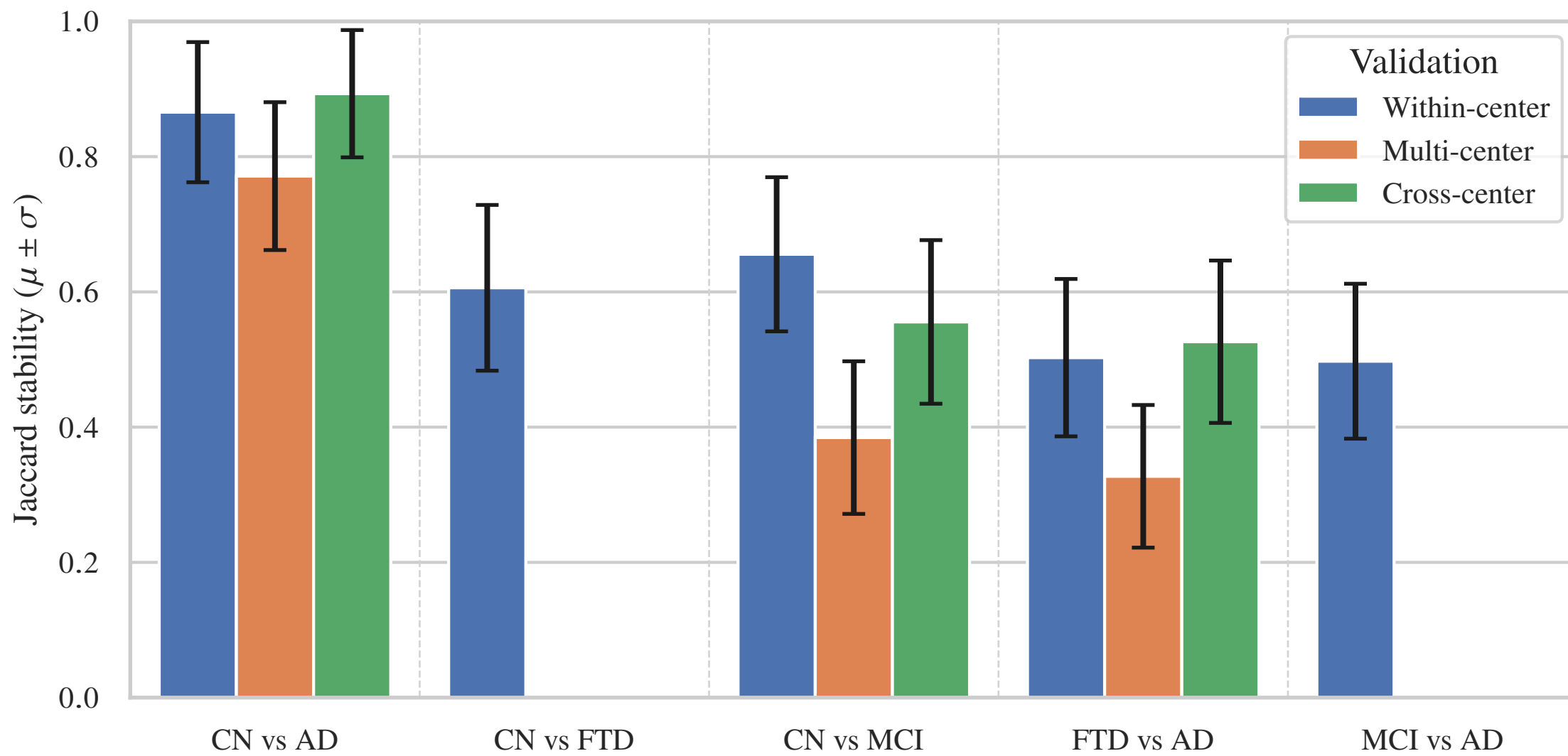

Figure S4: Jaccard similarity of top-10 feature sets (ranked by mean absolute SHAP value on the held-out test fold) across repeated cross-validation runs, shown for each diagnostic contrast and validation strategy (within-center, multi-center, and cross-center). For each repetition pair, the Jaccard index was computed as the size of the intersection divided by the size of the union of the two top-10 features, ranging from 0 (no overlap) to 1 (identical sets). Bars show the mean Jaccard similarity across all pairwise repetition comparisons; error bars indicate standard deviation from the mean. Absent bars indicate validation strategies not available for a given contrast.

10.4 Feature importance under downsampled balanced conditions

To verify that the neurophysiological signatures identified by SHAP analysis in the main analysis were not driven by imbalances in sample size across diagnostic categories and acquisition centers, we replicated the feature importance analysis using the same downsampled balanced datasets described in Section 10.1, in which an equal number of observations (n = 16) was drawn per diagnostic and center combination prior to model training.

The resulting SHAP profiles are presented in Supplementary material Figure S5. Feature importance patterns were highly consistent with those reported in the main analysis (Figure 5). For CN vs AD, the two opposing neurophysiological dynamics remained the dominant discriminative signature: alpha-band degradation — led by the temporal variability of alpha power ($\alpha_{\mu,\sigma}$) and alpha permutation entropy ($PE\alpha_{\sigma,\mu}$) — and concurrent elevation of slow-wave theta and delta features, with $|\theta|_{\sigma,\mu}$ retaining its rank as the single most influential feature. The directionality of all top features was unchanged relative to the full-sample analysis. For CN vs FTD, beta-band power and beta-band connectivity ($wSMI\beta$) continued to dominate, with median spectral frequency ($MSF_{\mu,\sigma}$) appearing somewhat more prominently than in the full-sample analysis, consistent with the spectral slowing expected in this contrast. For CN vs MCI, the heterogeneous and attenuated profile was preserved, with gamma-band variability ($\gamma_{\mu,\sigma}$) again leading, accompanied by delta and alpha entropy features; beta-band connectivity features appeared with slightly greater weight under balanced sampling, suggesting a modest contribution that may have been partially obscured by class imbalance in the full dataset. For FTD vs AD and MCI vs AD, the connectivity- and complexity-driven profiles described in the main text were likewise preserved, with delta and theta features maintaining their leading role in the MCI-to-AD transition.

medRxiv preprint doi: https://doi.org/10.64898/2026.07.28.26359133; this version posted July 29, 2026. The copyright holder for this preprint (which was not certified by peer review) is the author/funder, who has granted medRxiv a license to display the preprint in perpetuity. It is made available under a CC-BY 4.0 International license .

Contrast	Validation	Jaccard ($\mu \pm \sigma$)
	Within-center	0.87 ± 0.10
CN vs AD	Multi-center	0.77 ± 0.11
	Cross-center	0.89 ± 0.09
CN vs FTD	Within-center	0.61 ± 0.12
	Within-center	0.66 ± 0.11
CN vs MCI	Multi-center	0.38 ± 0.11
	Cross-center	0.56 ± 0.12
	Within-center	0.50 ± 0.12
FTD vs AD	Multi-center	0.33 ± 0.11
	Cross-center	0.53 ± 0.12
MCI vs AD	Within-center	0.50 ± 0.11

Table S1: Jaccard similarity of top-10 feature sets (ranked by mean absolute SHAP value on the held-out test fold) across repeated cross-validation runs, shown for each diagnostic contrast and validation strategy (within-center, multi-center, and cross-center). For each repetition pair, the Jaccard index was computed as the size of the intersection divided by the size of the union of the two top-10 features, ranging from 0 (no overlap) to 1 (identical sets). Table shows the mean and standard deviation of the Jaccard similarity across all pairwise repetition comparisons.

Across all contrasts, absolute SHAP magnitudes were modestly reduced relative to the full-sample analysis, consistent with the smaller training set sizes available under downsampling. Taken together, these results confirm that the disease-specific neurophysiological signatures identified in the main analysis are not artefacts of class or center imbalance, but reflect genuine and robust patterns in the EEG data.

10.5 Extended EEG preprocessing

All preprocessing was performed offline using a fully automated pipeline implemented in **MNE-Python** and the **NICE** framework, following procedures established for multi-center clinical EEG harmonization Sitt et al., 2014; Engemann et al., 2018. The pipeline was applied identically to all datasets without site-specific tuning.

10.5.1 Channel selection

Non-scalp and non-EEG channels were excluded, including electrooculographic (EOG), electromyographic (EMG), external reference, and system status channels. Only scalp EEG channels were retained for further processing.

10.5.2 Filtering

Signals were band-pass filtered between **0.5–45 Hz** using zero-phase IIR Butterworth filters. Implementation consisted of a **4th-order high-pass filter at 0.5 Hz** followed by an **8th-order low-pass filter at 45 Hz**. Both filters were applied with forward–backward (filtfilt) correction to eliminate phase distortion.

10.5.3 Resampling and trimming

All recordings were **resampled to 250 Hz** to ensure comparability of frequency-dependent markers across acquisition systems. The initial **0.8 s of each recording** were removed to exclude start-up transients.

10.5.4 Epoching

Continuous data were segmented into **0.8-s epochs** with **50% overlap**, providing stable estimation for spectral and information-theoretic markers.

10.5.5 Adaptive artifact rejection

Artifact detection followed a four-step procedure, applied sequentially to channels and epochs Engemann et al., 2018. Each step operated on peak-to-peak amplitude or variance statistics computed directly from the epoched data.

medRxiv preprint doi: https://doi.org/10.64898/2026.07.28.26359133; this version posted July 29, 2026. The copyright holder for this preprint (which was not certified by peer review) is the author/funder, who has granted medRxiv a license to display the preprint in perpetuity.
It is made available under a CC-BY 4.0 International license .

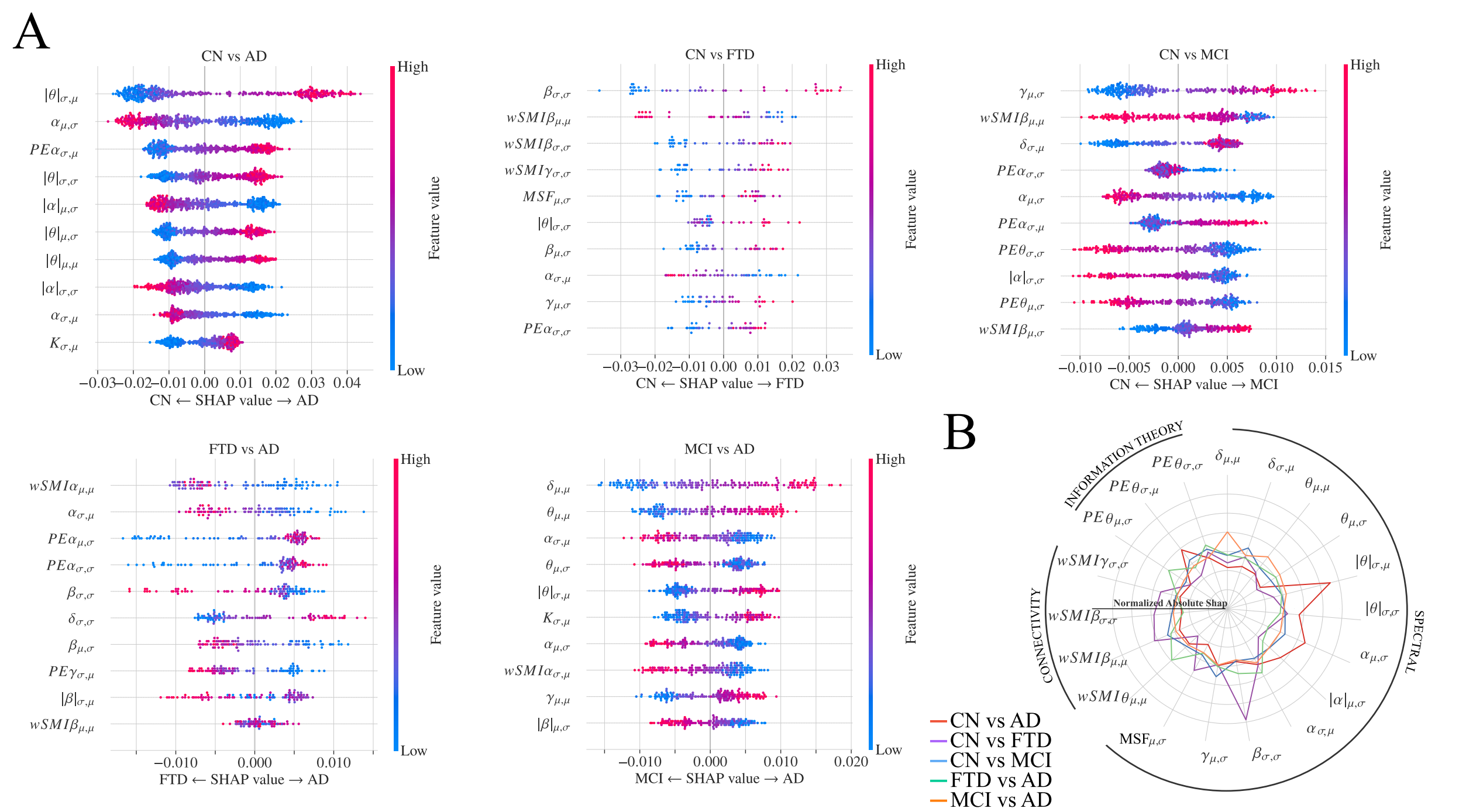

Figure S5: **Feature importance analysis using SHAP values under downsampled balanced conditions (n =** 16 **per diagnostic and center)**. Layout and notation follow Figure 5 of the main text. (A) Beeswarm plots showing the top-10 most influential EEG features for each binary classification task, with SHAP values averaged across all validation schemes and 500 repeated downsampling-plus-cross-validation iterations. Each point represents a single prediction; horizontal position indicates the SHAP contribution magnitude and direction, and color reflects the feature value (blue: low; red: high). Features are ordered by mean rank of absolute SHAP values. (B) Polar plot summarising normalised absolute SHAP values for features appearing in the top 5 of any classification task, with color-coded lines corresponding to each diagnostic contrast. Feature importance patterns are highly consistent with the full-sample analysis (Figure 5), confirming that the identified neurophysiological signatures are robust to class and center imbalance. Modest reductions in absolute SHAP magnitudes reflect the smaller training set sizes inherent to the downsampling procedure.

1. **Channel rejection by peak-to-peak range**. For each channel, the range (maximum minus minimum) was computed within every epoch. Channels were marked as bad when the number of epochs with a range exceeding **100 µV** was greater than **50% of all epochs**. This criterion identifies sensors with recurrent large-amplitude fluctuations across time.
2. **Channel rejection by variance outliers**. Channel variances were estimated across all samples, and an iterative Z-score procedure was applied using an outlier detection mechanism. Channels whose variance exceeded a threshold of **4 standard deviations** from the median were flagged as bad, with up to **4 iterations** to remove local outliers before recomputation.
3. **Epoch rejection by channel contamination**. For each epoch, the proportion of channels whose peak-to-peak range exceeded **100 µV** was calculated. Epochs were rejected when more than **10% of channels** violated this threshold, ensuring that transient artifacts affecting multiple sensors were removed.
4. **Channel rejection by high-frequency variance**. To capture residual muscle or line-noise contamination, data were high-pass filtered at **25 Hz** (4th-order Butterworth). Channels with abnormally large standard deviation in this high-frequency band were detected using the same iterative Z-score procedure (**4 SD**, **max 4 iterations**).

The procedure was terminated if all channels or all epochs were flagged as bad at any intermediate step. The final set of bad channels was the union of those detected across the three channel-level criteria.

10.5.6 Inclusion criteria

Recordings were retained only if at least **200 artifact-free epochs** remained and more than **70% of original EEG channels** were usable after rejection. All recordings passed inclusion criteria.

medRxiv preprint doi: https://doi.org/10.64898/2026.07.28.26359133; this version posted July 29, 2026. The copyright holder for this preprint (which was not certified by peer review) is the author/funder, who has granted medRxiv a license to display the preprint in perpetuity. It is made available under a CC-BY 4.0 International license .

10.5.7 Re-referencing and interpolation

Data were re-referenced to the **average reference** computed from all non-rejected channels. Bad channels were reconstructed using **spherical spline interpolation** to preserve the original montage geometry.

10.5.8 Implementation notes

All steps were executed automatically with fixed parameters across centers to avoid site-specific optimization. The pipeline was applied prior to feature extraction without access to diagnostic labels, ensuring independence between preprocessing and subsequent machine-learning analyses.

10.5.9 Overall Data Quality (ODQ) analysis

To verify that the preprocessed EEG recordings did not exhibit systematic differences in signal quality across diagnostic groups, which could confound downstream feature extraction, we computed the Overall Data Quality (ODQ) index for each EEG recording following the methodology proposed by (Zhao et al., 2023). ODQ quantifies the proportion of artifact-free signal segments and is derived from four complementary detection methods applied to the epoched data (0.8 seconds). The four methods flag windows containing: (i) constant or flat signals (ONS; detected via standard deviation and median absolute deviation thresholds below 10^{-10}); (ii) high-amplitude transients (OHA; detected via an absolute amplitude threshold of 150 µV and a z-scored robust standard deviation threshold); (iii) high-frequency noise (OFN; detected via the noise-to-signal ratio relative to the power-line frequency cutoff, with both absolute and z-scored thresholds); and (iv) low inter-channel correlation (OLC; detected when the maximum Pearson correlation between a channel and all others fell below 0.6). A window was classified as good quality only when it passed all four criteria, and ODQ was expressed as the percentage of good windows over the total number of windows.

To test whether ODQ differed systematically across diagnostic categories, pairwise comparisons between all condition pairs (CN, MCI, AD, FTD) were performed using two-sided Mann-Whitney U tests, both globally across all datasets pooled and separately within each of the six participating centers.

No significant differences in ODQ were found between any pair of diagnostic conditions, either in the pooled analysis (Figure S6 or within individual datasets (Figures S7). These results confirm that the preprocessed recordings are balanced in signal quality across diagnostic groups and that observed differences in EEG features are unlikely to be attributable to differential artifact contamination.

10.6 Extended EEG feature extraction

EEG biomarkers were computed using the NICE Python library following the framework of Sitt et al. (2014) and Engemann et al. (2018). Feature extraction was applied identically to all centers and exclusively on preprocessed data, without access to diagnostic labels.

10.6.1 Marker families

Spectral markers. Power spectral density was estimated for each channel and epoch using Welch's method. Features were computed in canonical frequency bands: δ (1.25–4 Hz), θ (4–8 Hz), α (8–13 Hz), β (13–30 Hz), and γ (30–45 Hz). Derived descriptors included absolute and relative band power, median spectral frequency, spectral edge (90/95%), and spectral entropy. It's worth noticing that the δ band is calculated from 1.25Hz because epochs with 0.8 seconds length make it not possible to calculate lower frequencies.

Information-theoretic markers. Signal irregularity was quantified using permutation entropy (PE) with embedding dimension $m = 3$ and band-specific delays τ matched to the center frequency of each band. Kolmogorov complexity (K) was estimated to capture algorithmic compressibility of the time series.

Connectivity markers. Inter-electrodes coupling was assessed using weighted symbolic mutual information (wSMI). After symbolic discretization of the signals, mutual information was computed for all channel pairs, providing a nonlinear connectivity measure designed to attenuate spurious correlations due to volume conduction.

A complete list of the 23 biomarkers across the 3 families is provided in Supplementary Table S2.

10.6.2 Spatial and temporal aggregation

For each biomarker B, values were first computed at the level of individual channels and epochs. To obtain center-agnostic representations, two complementary reductions were applied:

medRxiv preprint doi: https://doi.org/10.64898/2026.07.28.26359133; this version posted July 29, 2026. The copyright holder for this preprint (which was not certified by peer review) is the author/funder, who has granted medRxiv a license to display the preprint in perpetuity.
It is made available under a CC-BY 4.0 International license .

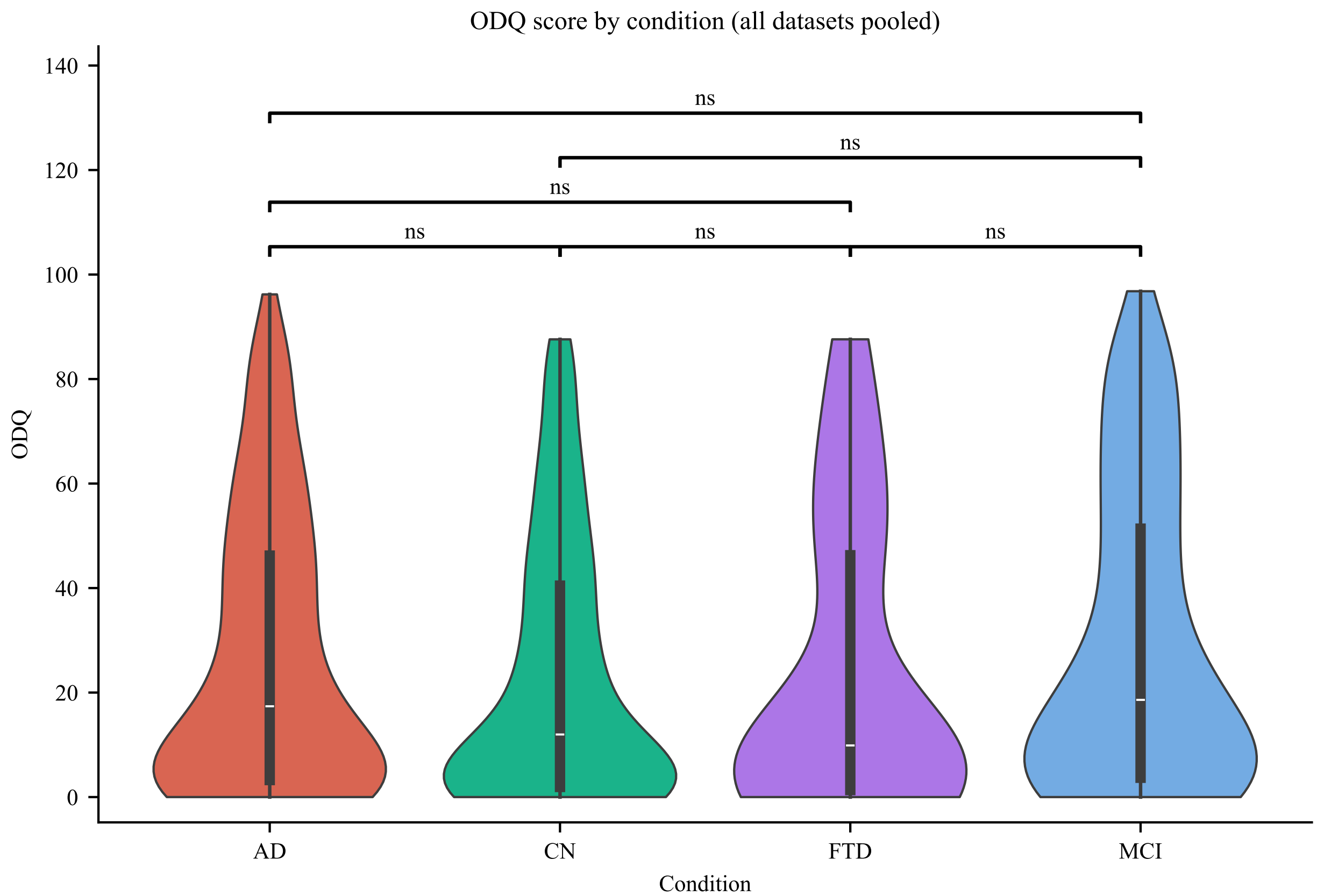

Figure S6: **Distribution of the Overall Data Quality (ODQ) score across diagnostic conditions across all centers**: Each violin shows the full distribution of ODQ scores; Pairwise significance labels reflect Mann-Whitney, "ns" denotes no significant difference. No condition pair showed a statistically significant difference in ODQ.

- **Spatial summaries across channels**
  - Channel mean: reflects overall magnitude of the marker on the scalp.
  - Channel standard deviation: reflects **global field power**, i.e., the spatial heterogeneity of the marker across electrodes.
- **Temporal summaries across epochs**
  - 80% trimmed mean: captures typical activity while reducing sensitivity to residual artifacts.
  - Standard deviation: quantifies **temporal variability** of the marker within the recording.

Combining these dimensions yielded four descriptors for each biomarker:

- μ/μ: mean across channels and trimmed mean across epochs (average activity),
- σ/μ: standard deviation across channels and trimmed mean across epochs (global field power),
- μ/σ: mean across channels and standard deviation across epochs (temporal variance),
- σ/σ: standard deviation across channels and epochs (joint spatiotemporal variability).

This dual aggregation produced **92 feature subtypes** (23 biomarkers × 4 descriptors) used as input to the classification models.

10.6.3 Implementation

All computations were performed with fixed parameters across centers. Spectral, information-theoretic, and connectivity features were extracted from identical epochs defined in the preprocessing stage. No feature selection or parameter tuning was performed prior to model training to preserve independence between feature extraction and classification.

medRxiv preprint doi: https://doi.org/10.64898/2026.07.28.26359133; this version posted July 29, 2026. The copyright holder for this preprint (which was not certified by peer review) is the author/funder, who has granted medRxiv a license to display the preprint in perpetuity.
It is made available under a CC-BY 4.0 International license .

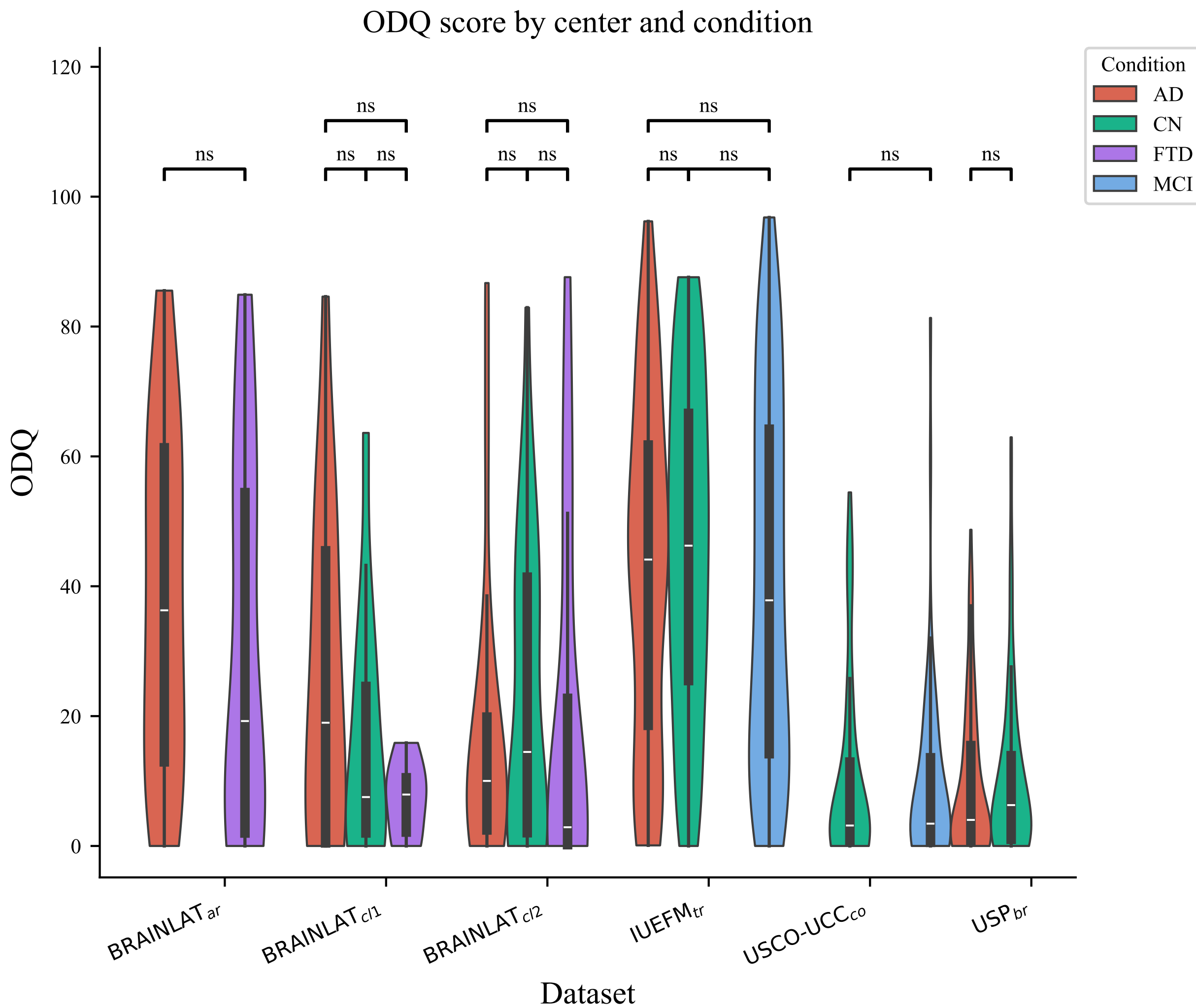

Figure S7: **Distribution of the Overall Data Quality (ODQ) score stratified by center and diagnostic condition:** Each x position corresponds to a specific center, and violins are colored by diagnostic conditions inside each center. Pairwise significance brackets reflect Mann-Whitney U tests between condition within each center; "ns" denotes no significant difference. No center exhibited a significant ODQ difference across diagnostic groups.

10.7 Extended evaluation, calibration, and brain–behavior association analyses

Model performance across all validation folds was evaluated using the area under the receiver operating characteristic curve (ROC-AUC), a threshold-independent metric quantifying the discriminative ability between diagnostic categories.

To assess the reliability of the predicted probabilities, we compared the uncalibrated and calibrated model outputs (Figure 1 Calibration) to determine i) whether the model was intrinsically well-calibrated (Cal_{err}) and ii) whether calibration procedures improved the alignment between predicted and true outcome frequencies (Cal_{Δ}). Model calibration was performed using CalibratedClassifierCV Python class provided by the scikit-learn library with Platt scaling (sigmoid calibration) and five-fold cross-validation. This procedure fits a sigmoid function to the model's decision scores, transforming them into probability estimates that more closely reflect empirical likelihoods. For instance, in a well-calibrated model, among all cases predicted with a 60% probability of being MCI, approximately 60% should actually belong to that category.

Calibration, however, cannot compensate for a poorly discriminative classifier: if the base model fails to separate diagnostic groups, no post-hoc transformation can yield meaningful probabilities. Furthermore, even for accurate models,

medRxiv preprint doi: https://doi.org/10.64898/2026.07.28.26359133; this version posted July 29, 2026. The copyright holder for this preprint (which was not certified by peer review) is the author/funder, who has granted medRxiv a license to display the preprint in perpetuity. It is made available under a CC-BY 4.0 International license .

calibration may remain imperfect when the decision function is highly non-monotonic or when score distributions are discrete, as often occurs in tree-based ensembles such as Random Forests.

To assess whether the observed classification performance exceeded chance level, we implemented a two-stage statistical validation framework (Figure 1 Permutation Test).

Stage 1 — Repeated cross-validation. To enhance robustness, we conducted 500 repetitions of k-fold cross-validation. At each repetition, the dataset was randomly partitioned into new folds, and model predictions were obtained exclusively from out-of-fold test samples, ensuring independence between training and testing data. This procedure yielded a distribution of 500 AUC values for each validation strategy, classification task and center, representing the variability and stability of model performance across split iterations.

Stage 2 — Permutation testing. To evaluate statistical significance, we performed 500 label permutations per task using the same cross-validation structure. In each permutation, diagnostic labels were randomly shuffled while preserving data structure and class proportions, thereby generating a null distribution of performance metrics expected under the assumption of no true association between EEG features and diagnostic categories.

Statistical inference (Figure 1 Performance Distributions). Model performance was then assessed using the difference of means between the true labels AUCs and the permuted labels AUCs. We computed the significance level using the proportion of permuted AUCs greater than the observed median of real AUC as the empirical p_{val}, thus estimating the probability that a model trained on data with no true association between markers and diagnostic categories could achieve comparable performance. Finally, we corrected the p_{val} wit FDR using the Benjamini–Hochberg procedure across all obtained p_{val}.

To quantify the consistency of individual-level model predictions across validation strategies, we analyzed correlations between predicted probabilities obtained under within-center, multi-center, and cross-center validation. For each diagnostic contrast, predictions were extracted exclusively from out-of-fold test samples to avoid information leakage. Subject-level predicted probabilities were averaged across repetitions prior to correlation analysis.

Spearman rank correlations were computed between each pair of validation strategies (within vs multi, within vs cross, and multi vs cross) to assess the stability of subject ranking across training conditions. Correlations were evaluated separately for each diagnostic contrast and, where applicable, within individual centers. Statistical significance was assessed using the p_{val} obtained from the Spearman rank statistic, and FDR correction using the Benjamini–Hochberg procedure was applied across all correlations to account for multiple comparisons.

Associations between model predictions and cognitive measures were computed using residualized Spearman correlations. For each subject, predicted probabilities were extracted from out-of-fold test predictions under within-center, multi-center, or cross-center validation and averaged across repeated cross-validation iterations when applicable. cognitive measures included measures of global cognition, memory, language, attention, and executive function, depending on availability at each center.

To control for demographic confounds, both predicted probabilities and cognitive scores were independently residualized with respect to age, sex, and years of education using linear regression and Spearman rank correlations were computed between residuals using the python package *Pingouin* (Vallat, 2018), which comes with a build-in function to do this (partial_corr). Analyses were conducted separately for each diagnostic contrast and validation strategy. Statistical significance was evaluated with FDR correction using the Benjamini–Hochberg procedure per validation strategy, cognitive measures and contrasts. In the correlation heatmaps, all coefficients are displayed, with color indicating correlations that survived FDR correction.

Finally, to identify the EEG features most discriminative across diagnostic groups and to provide interpretable insights into model decisions, we applied SHAP (SHapley Additive exPlanations) analysis (S. Lundberg & Lee, 2017; S. M. Lundberg et al., 2020) (Figure 1 SHAP Values Feature Importance). SHAP values quantify the magnitude and direction of each feature's contribution to individual predictions, thereby enabling both global feature importance ranking and local interpretability. This approach enhances transparency by revealing which EEG characteristics most strongly influence diagnostic classification.

Model interpretability was assessed using SHAP values computed in an out-of-fold manner alongside the cross-validation procedure. For each fold, models were trained on the training partition, and SHAP values were estimated on the corresponding held-out test partition using a permutation-based explainer. The background distribution was defined using the training data of each fold, ensuring that explanations were computed without information leakage.

This procedure yielded, for each validation strategy, diagnostic contrast, sample, and feature, a distribution of SHAP values across 500 repetitions. SHAP values were subsequently averaged across repetitions for each sample and classification contrast to obtain robust estimates of feature importances.

medRxiv preprint doi: https://doi.org/10.64898/2026.07.28.26359133; this version posted July 29, 2026. The copyright holder for this preprint (which was not certified by peer review) is the author/funder, who has granted medRxiv a license to display the preprint in perpetuity.
It is made available under a CC-BY 4.0 International license .

To visualize the effect of individual markers, we examined the ten features with the highest absolute mean SHAP values for each classification task, analyzing how their magnitude related to SHAP contribution—that is, whether higher or lower feature values increased the likelihood of predicting one class over another. Subsequently, the five most influential features per task where all pooled together in a radar plot to facilitate cross-task comparison.

**Notation**	**Marker**	**Conceptual Family**
K	Kolmogorov complexity	Complexity
PE_α	Permutation entropy alpha band	Information Theory
PE_β	Permutation entropy beta band	Information Theory
PE_θ	Permutation entropy theta band	Information Theory
PE_γ	Permutation entropy gamma band	Information Theory
α	Energy alpha PSD (8–13 Hz)	Spectral
β	Energy beta PSD (13–30 Hz)	Spectral
δ	Energy delta PSD (1–4 Hz)	Spectral
γ	Energy gamma PSD (30–40 Hz)	Spectral
θ	Energy theta PSD (4–8 Hz)	Spectral
$\|\alpha\|$	Energy Normalized alpha PSD (8–13 Hz)	Spectral
$\|\beta\|$	Energy Normalized beta PSD (13–30 Hz)	Spectral
$\|\delta\|$	Energy Normalized delta PSD (1–4 Hz)	Spectral
$\|\gamma\|$	Energy Normalized gamma PSD (30–40 Hz)	Spectral
$\|\theta\|$	Energy Normalized theta PSD (4–8 Hz)	Spectral
MSF	Median Power Frequency	Spectral
SEF_{90}	Spectral edge 90	Spectral
SEF_{95}	Spectral edge 95	Spectral
SE	Spectral entropy	Spectral
$wSMI_\alpha$	Weighted symbolic mutual information alpha band	Connectivity
$wSMI_\beta$	Weighted symbolic mutual information beta band	Connectivity
$wSMI_\theta$	Weighted symbolic mutual information theta band	Connectivity
$wSMI_\gamma$	Weighted symbolic mutual information gamma band	Connectivity

Table S2: Summary of the EEG markers considered in the study taken from (Sitt et al., 2014; Engemann et al., 2018). The notation column shows the abbreviated symbol used in analyses, the marker column provides the full description (including frequency ranges for spectral measures), and the conceptual family indicates the theoretical category of each marker.

medRxiv preprint doi: https://doi.org/10.64898/2026.07.28.26359133; this version posted July 29, 2026. The copyright holder for this preprint (which was not certified by peer review) is the author/funder, who has granted medRxiv a license to display the preprint in perpetuity.
It is made available under a CC-BY 4.0 International license .

Contrast	Center	Correlation	r	p-value
CN vs AD	$BRAINLAT_{cl1}$	Multi vs Cross	0.94	0.00
		Within vs Cross	0.68	0.00
		Within vs Multi	0.83	0.00
	$IUEFM_{tr}$	Multi vs Cross	0.89	0.00
		Within vs Cross	0.76	0.00
		Within vs Multi	0.95	0.00
	$BRAINLAT_{cl2}$	Multi vs Cross	0.96	0.00
		Within vs Cross	0.80	0.00
		Within vs Multi	0.88	0.00
	USP_{br}	Multi vs Cross	0.93	0.00
		Within vs Cross	0.83	0.00
		Within vs Multi	0.95	0.00
CN vs MCI	$IUEFM_{tr}$	Multi vs Cross	0.33	0.00
		Within vs Cross	0.17	0.02
		Within vs Multi	0.97	0.00
	$USCO-UCC_{co}$	Multi vs Cross	0.44	0.00
		Within vs Cross	-0.01	0.89
		Within vs Multi	0.76	0.00
FTD vs AD	$BRAINLAT_{ar}$	Multi vs Cross	0.69	0.00
		Within vs Cross	-0.05	0.78
		Within vs Multi	0.62	0.00
	$BRAINLAT_{cl2}$	Multi vs Cross	0.13	0.38
		Within vs Cross	-0.24	0.09
		Within vs Multi	0.88	0.00

Table S3: Subject level predicted probabilities correlations between validation strategies by diagnostic contrast and Center.
